# Bayesian model selection reveals biological origins of zero inflation in single-cell transcriptomics

**DOI:** 10.1186/s13059-020-02103-2

**Published:** 2020-07-27

**Authors:** Kwangbom Choi, Yang Chen, Daniel A. Skelly, Gary A. Churchill

**Affiliations:** 1 The Jackson Laboratory, 600 Main Street, Bar Harbor, 04609 ME USA; 2 University of Michigan, 500 South State Street, Ann Arbor, 48109 MI USA

**Keywords:** Single-cell RNA sequencing, Zero inflation, Bayesian model selection, Cell heterogeneity, Gene expression stochasticity

## Abstract

**Background:**

Single-cell RNA sequencing is a powerful tool for characterizing cellular heterogeneity in gene expression. However, high variability and a large number of zero counts present challenges for analysis and interpretation. There is substantial controversy over the origins and proper treatment of zeros and no consensus on whether zero-inflated count distributions are necessary or even useful. While some studies assume the existence of zero inflation due to technical artifacts and attempt to impute the missing information, other recent studies argue that there is no zero inflation in scRNA-seq data.

**Results:**

We apply a Bayesian model selection approach to unambiguously demonstrate zero inflation in multiple biologically realistic scRNA-seq datasets. We show that the primary causes of zero inflation are not technical but rather biological in nature. We also demonstrate that parameter estimates from the zero-inflated negative binomial distribution are an unreliable indicator of zero inflation.

**Conclusions:**

Despite the existence of zero inflation in scRNA-seq counts, we recommend the generalized linear model with negative binomial count distribution, not zero-inflated, as a suitable reference model for scRNA-seq analysis.

## Results

### Why are there so many zeros?

**Fig. 1:**
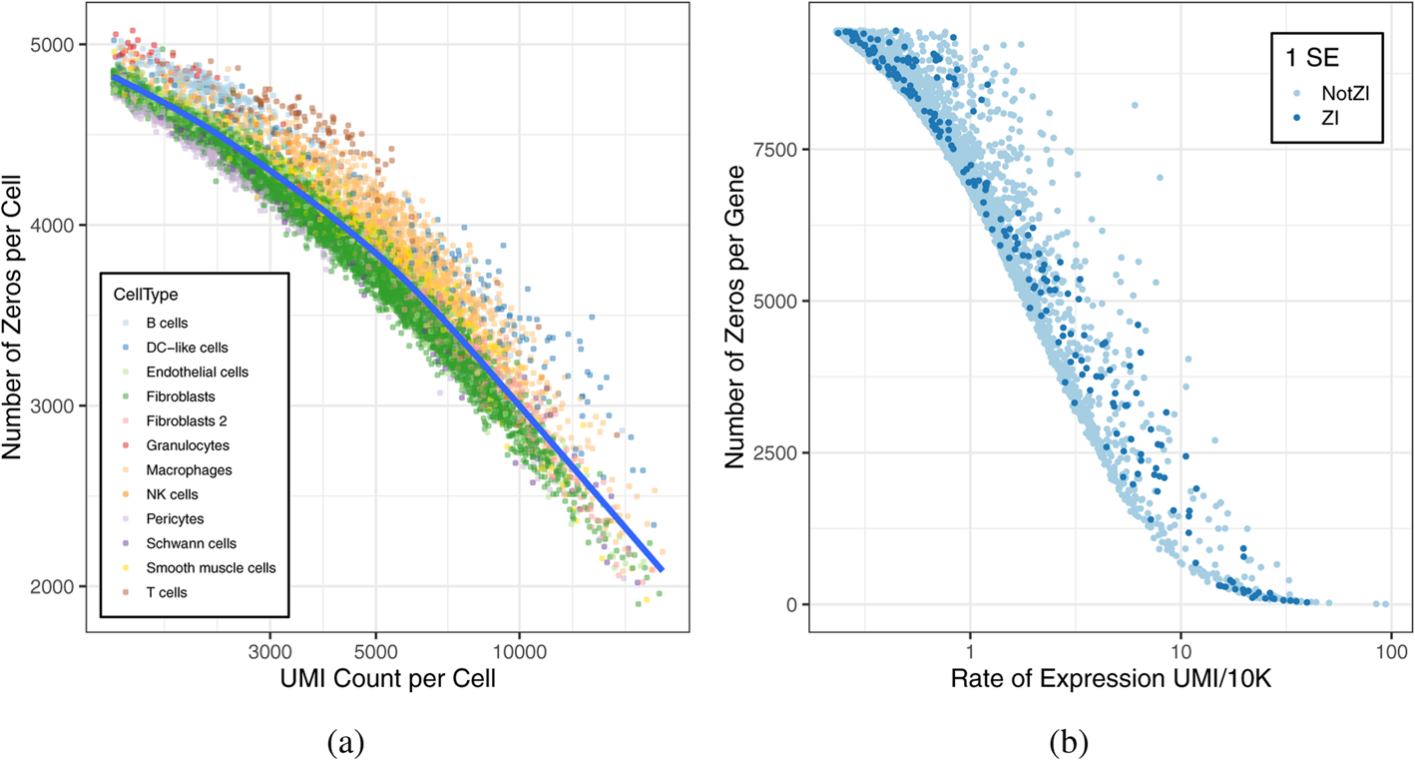
Factors that determine the number of zeros in scRNA-seq data. **a** Total UMI counts per cell, which range from 746 to 17,302 with average 3819 UMIs per cell, are plotted against the number of zeros per cell. Color coding indicates the individual cell types as determined by data-driven clustering. The proportion of variance in the number of zeros that is explained by the total UMI count per cell (*R*^2^= 0.947) was computed based on fitting a loess regression to the data (blue curve). **b** The per-gene rates of expression (*μ*_*g*_), which range from 0.23 to 97.4 with average 1.51 UMI/10K, are plotted against the number of zeros per gene. Genes that were identified as zero-inflated by scRATE (1 SE) are indicated in dark blue

The most important factor that determines the number of zeros in scRNA-seq data is the sequencing depth (total UMI count) per cell. In the heart data, this ranges from 746 to 17,302 UMIs per cell after filtering to 5515 genes in order to remove genes with non-zero UMI counts in less than 10% of cells (Fig. 1a and Additional file 1: Fig. S1a and S1b). Clearly, if the total UMI count in a cell is less than the number of genes, some genes will have zero counts. Sequencing depth explains 95% of variation in the number of zeros per cell (*R*^2^= 0.945, *p*<2.2e−16). While our focus here is on droplet-based scRNA-seq which yields low total UMI counts on very large numbers of cells, the number of zeros is also largely determined by read depth for single-cell sequencing platforms (e.g., Fluidigm C1) that produce deeper coverage of smaller numbers of cells. For example, Bacher et al.’s [20] datasets have *R*^2^= 0.384. To account for variation in sequencing depth, we incorporate log of the total UMI count per cell as an offset in the generalized linear model (see the “Methods” section). An offset can be thought of as a covariate for which the regression parameter is fixed and not estimated. Hafemeister and Satija [21] have recently shown that treating the offset as a covariate with an estimated coefficient leads to overfitting. The effect of including an offset is simply to rescale the data from a count to a rate. In addition to the overfitting problem, the use of a fixed regression coefficient provides a consistent interpretation of the estimated model parameters as a rate of expression. For example, if we set the offset to log(total UMI count per cell/ 10e5), the mean parameters from any of the GLMs (P, NB, ZIP, or ZINB) can be interpreted as the expected gene-specific UMI count per 10,000 total UMI.

The second most important factor in determining the number of zeros is the per-gene average rate of expression. In the heart data, the rate of expression varies from 0.23 to 97.4 UMI/10K across 5515 genes (Fig. 1b and Additional file 1: Fig. S1c). In general, genes with lower rates of expression will have a higher frequency of zeros. We compared the expected number zeros for each gene, assuming a Poisson model with matching gene-specific rates of expression and cell-specific offsets, to the observed numbers of zeros in the heart data (Additional file 1: Fig. S1d). We see that many genes have “extra” zeros, in some cases with thousands of zeros over expectation. Presumably, these genes have count distributions that are overdispersed (NB), zero-inflated (ZIP), or both (ZINB).

### Model selection can identify genes exhibiting zero inflation

In order to identify genes with zero inflation, we implemented a Bayesian model selection criterion—the expected log predictive density (ELPD) [16]—in our software package, scRATE (https://github.com/churchill-lab/scRATE). The ELPD score estimates out-of-sample predictive accuracy of four statistical models (P, ZIP, NB, or ZINB). It penalizes both underfitted and overfitted models. It examines all of the data, including non-zero counts, to provide a more complete evaluation of the count distributions than approaches that focus only on the zeros [8, 9]. scRATE uses leave-one-out cross-validation, which provides a standard error (SE) to quantify uncertainty in the estimated ELPD scores. The four models being compared have varying levels of complexity (P ≺NB, P ≺ZIP, NB ≺ZINB, and ZIP ≺ZINB), and in order to ensure that a more complex model is selected only when the ELPD is substantially better, we require that the difference in ELPD between two models is greater than zero by a multiple of the SE (e.g., 0 SE, 1 SE, 2 SE, or 3 SE). In addition to the model selection criterion, scRATE reports Bayesian parameter estimation and it can be used as a replacement for or as a complementary analysis tool along with standard GLM software.

To evaluate the true positive and false positive rates for detecting zero-inflated (ZI) genes—genes for which either the ZIP or ZINB model is selected—we simulated data similar to the heart data but with fixed levels of zero inflation ($\widehat {\pi }_{0}$) ranging from 0 to 90% and depth of sequencing at 10,000 UMIs/cell (*Simulation I* in the “Methods” section and Additional file 1: Fig. S2a). We applied scRATE to the simulated data using the 0, 1, and 2 SE thresholds (Table 1). scRATE has a high false positive rate at 0 SE, but at the 1 SE threshold, the false positive error rate falls below 0.05, and at the 2 SE threshold, false positives are controlled at a stringency suitable for multiple testing across genes. In addition, we simulated data with average sequencing depths up to 50,000 UMIs/cell—higher than most droplet scRNA-seq data—and observed a substantial improvement in power (Additional file 1: Fig. S2b). This suggests that deeper coverage may be beneficial for detecting ZI genes. We carried out additional simulations to examine performance of different thresholds as described in the “Methods” section (*Simulation II*) and Supplementary Materials (Additional file 1: Fig. S3). Having established that scRATE can detect ZI genes in simulated data, we next applied our model selection criterion to the heart data.

**Table 1:** Error rates and power of scRATE classification

Sequencing depth	Threshold		
	0 SE	1 SE	2 SE
(a)			
10k	0.2349_±0.0695_	0.0325_±0.0174_	0.0014_±0.0016_
50k	0.1837_±0.0557_	0.0206_±0.0159_	0.0009_±0.0016_
(b)			
10k	0.8116_±0.0365_	0.6152_±0.0312_	0.4641_±0.0160_
50k	0.8955_±0.0158_	0.7934_±0.0176_	0.7062_±0.0165_

Estimated false positive (FP, type I error) and true positive (TP, power) classification rates estimated from simulated data at average depth of 10,000 or 50,000 UMIs per cell. See *Simulation II* in the “Methods” section for details

### scRNA-seq data are zero-inflated for some genes

Townes et al. [8] and Svensson [9] have shown that the P or NB models, without zero inflation, are sufficient to capture technical variability of scRNA-seq data. It is still of interest to determine whether these models are flexible enough to also capture biological heterogeneity. In order to evaluate whether and how biological factors are contributing to zero inflation, we initially analyzed the heart data without considering any associated biological knowledge. We applied scRATE to each of 5515 genes to classify them according to their best fitting model (Table 2(a)) and to identify ZI genes. We found that for 1474 genes, the best model (0 SE) is one of the ZI options. Using more conservative thresholds, we found 220 genes (1 SE), 76 genes (2 SE), or 35 genes (3 SE) were best fit by a ZI model.

**Table 2:** scRATE classification of genes in the heart data

Threshold	Selected model			
	P	NB	ZIP	ZINB
(a)				
0 SE	1111	2930	525	949
1 SE	2112	3183	81	139
2 SE	2930	2509	5	71
3 SE	3445	2035	1	34
(b)				
0 SE	1523	2317	518	1105
1 SE	2733	2583	63	84
2 SE	3544	1913	3	3
3 SE	4000	1461	0	2
(c)				
0 SE	1118	2745	478	1094
1 SE	2107	3108	88	132
2 SE	2930	2432	5	68
3 SE	3420	1981	1	33

Genes were classified as one of four count models (P, NB, ZIP, or ZINB) using four levels of stringency (0 SE, 1 SE, 2 SE, or 3 SE). Table shows the number of genes in each category using a GLM with only the offset term to account for cell sequencing depth (a), using a GLM that also includes cell type as an explanatory covariate (b), and using a GLM that includes offset as well as a randomly shuffled cell type as a covariate (c). See Additional file 1: Tables S3 and S4 for the results with the mouse kidney and the human PBMC datasets

In order to evaluate the extent of under-calling of ZI genes by scRATE, we first down-sampled the data by randomly selecting subsets of cells and then repeated the model selection analysis (*Simulation III* in the “Methods” section and Additional file 1: Fig. S4). The number of ZI genes detected continues to increase with the number of cells even up to 10,000 cells. This suggests that the number of ZI genes detected, especially at the stringent 2 SE threshold, is an underestimate of the actual number of ZI genes. The ZI genes detected at 2 SE represent a lower bound on the number of high-confidence ZI genes that might be detected in a larger number of cells.

**Fig. 2:**
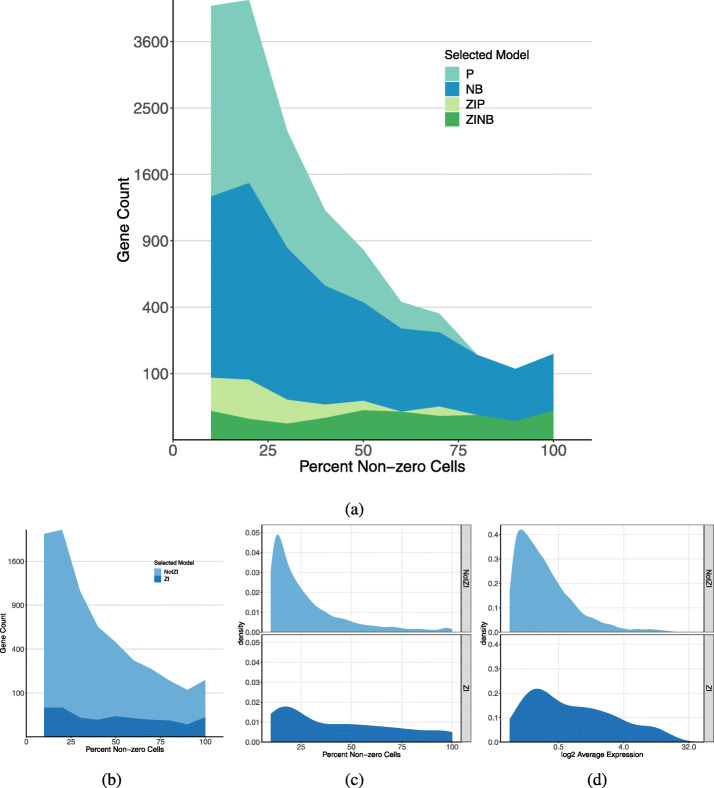
Classification of genes by scRATE using the threshold of 1 SE. **a** A density histogram shows the model selection for genes by scRATE as a function of percent non-zero cells. The ZI genes are uniformly distributed across the range, including genes with few zero counts. **b** Density histogram of scRATE classification collapsed to show only the ZI versus NotZI genes across percentages of non-zero cells. **c** Distribution of percent of cells with non-zero UMI counts for genes according to scRATE classification. **d** Distribution of average expression levels of genes according to scRATE classification. Also see Additional file 1: Fig. S12 for the results with the other (0, 2, and 3 SE) thresholds

It seems intuitive that ZI genes would have a higher proportion of zeros and lower average expression when compared to other genes [6]. However, our findings support the opposite conclusion (Fig. 1a and Additional file 1: Table S1). In our analysis of the heart data, ZI genes often have a lower proportion of zeros and higher rates of expression compared to genes that are not ZI (Fig. 2b–d). In cells where ZI genes are expressed, they exhibit higher average levels of expression compared to genes without zero inflation. The proportion of zeros is on average higher for NB genes compared to ZINB genes. The statistical test for zero inflation will have best power in cases where the gene is not expressed in some cell type(s), but when it is expressed, expression levels are high. When expression levels are low across all cell types, zero inflation is hard to distinguish from statistical sampling from a non-zero-inflated distribution. As a result, genes that are declared to be ZI using statistical testing are potentially biased toward higher levels of expression. Nonetheless, the number of zeros alone is not a good indicator of zero inflation; rather, one must consider the entire count distribution to establish that zero inflation is present.

### Most zero-inflated genes are due to variable expression rates across cell types

**Fig. 3:**
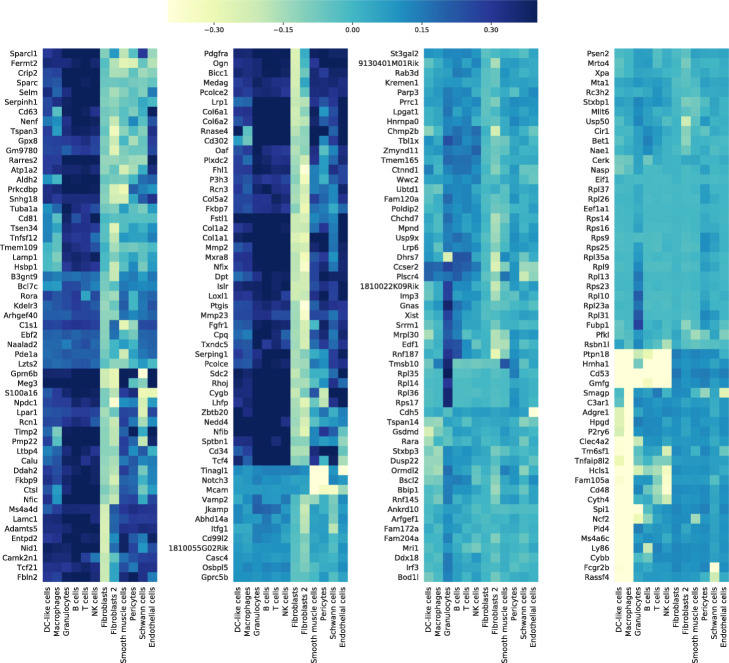
Zeros cluster within specific cell types. A bi-clustered heatmap of ZI genes (1 SE) by cell type shows that zeros occur more frequently in specific cell types. The color scale indicates the difference between the cell type-specific proportion of zeros and the mean proportion of zeros across all cells regardless of type. Light shading indicates cell types that have highest frequency of zero UMI counts. Dendrograms are shown in Additional file 1: Fig. S5

Skelly et al. [17] classified each cell in the heart data into one of 12 cell types by data-driven clustering and integration of previous biological knowledge. The annotated cell types are heterogeneous and include cell types that are similar to one another (e.g., macrophages and dendritic cells) and cell types that are very different (e.g., smooth muscle cells and B cells). If zero inflation is primarily due to technical dropout, we would expect to see zeros evenly distributed across cell types. When we examined the distribution of zeros across cells in the ZI genes, we found that they tended to cluster within certain cell types (Fig. 3). The rate of expression of a gene is a major factor driving the frequency of zeros, and for many genes, the rate of expression varies widely across cell types. This suggests that we should evaluate zero inflation after taking cell type-specific rates of expression into account.

To account for biological variation in expression rates, we introduced cell type as an explanatory variable in the GLM and recomputed the scRATE classification (Table 2(b)). After accounting for cell type, the number of zero-inflated genes drops markedly. Of the 76 genes that were originally classified as zero-inflated using the 2 SE thresholds, 72 are no longer classified as ZI, 3 genes remain ZI (*Xist, Rc3h2, Cir1*), and 3 genes become ZI after accounting cell type (*Prnp, Folr2, Tax1bp2*), and for one gene (*Mmp2*), the scRATE algorithm failed to converge when cell type was included in the model. Genes that are no longer ZI after accounting for cell type display variation in rates of expression across cell types, such as *Col1a2* which is expressed primarily in fibroblasts, or *Ptpn18* which is expressed primarily in immune cells (Additional file 1: Fig. S6).

In order to assess if this change in number of ZI genes was due to fitting the more complex model, we shuffled the cell type labels and repeated the scRATE classification. Results with the labels shuffled are similar to the scRATE classification without cell type (Table 2 (c)), demonstrating that the reduction in detected ZI genes is not due to a loss of power when including cell type as an explanatory variable.

While the majority of genes that were originally classified as ZI are no longer ZI after accounting for cell type, there are a handful of genes that remain or become ZI. Among them, *Xist* is an X chromosome silencing gene that is expected to be expressed only in female cells (Additional file 1: Fig. S7). The heart data represent a mixture of female and male cells. We were able to unambiguously classify 63% of cells in silico as female or male in origin based on the presence of UMIs associated with female-specific *Xist* or with the Y-chromosome gene *Ddx3y*. scRATE classifies *Xist* as a zero-inflated gene at all thresholds up to 2 SE, but *Ddx3y* is classified as NB and is only classified as a ZI gene at 0 SE after adjusting for cell type. After accounting for sex as an explanatory variable, in the subset of cells where we could establish sex, these genes are no longer ZI.

**Fig. 4:**
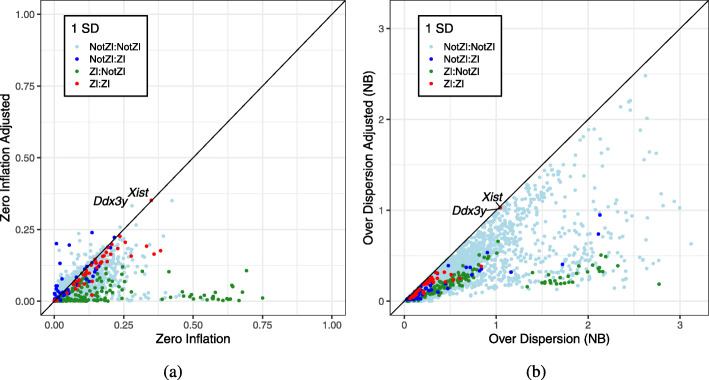
Effect of accounting for cell type on estimated zero inflation and overdispersion. **a** The scatterplot shows estimated zero inflation $\widehat {\pi }_{0}$ before and after including cell type in the GLM with ZINB error model. Color coding indicates the ZI classification of genes (1 SE) before and after accounting for cell type. The red point (ZI:ZI) at 0.3 on the diagonal is *Xist*. The light blue point (NotZI:NotZI) to the right is *Ddx3y*. **b** The scatterplot shows the estimated overdispersion $\hat {r}$ before and after including cell type in the GLM with NB error model

We fit a ZINB model to all genes and compared the estimated proportion of zero inflation (*π*_0_), with and without cell type in the GLM (Fig. 4a). For most genes, $\widehat {\pi }_{0}$ decreases. This is most evident among the genes that were classified as ZI before the adjustment and are no longer ZI after. For genes that remain ZI after accounting for cell type, there is little change in $\widehat {\pi }_{0}$. For a handful of genes, including those that become ZI only after accounting for cell type, $\widehat {\pi }_{0}$ increases. These changes in $\widehat {\pi }_{0}$ are consistent with expectations from the model selection analysis. Genes with higher values of $\widehat {\pi }_{0}$ are more likely to be classified as ZI.

Next, we fit the NB model to all genes and compared the estimated overdispersion ($\hat {r}$) with and without cell type in the GLM (Fig. 4b). Accounting for cell type consistently reduces $\hat {r}$, and the effects on different classes of ZI genes are similar to those for the $\widehat {\pi }_{0}$ values. Thus, the overdispersion parameter of the NB model is able to identify much of the same heterogeneity that we are capturing with the ZINB model.

### Estimated zero inflation is not a reliable indicator of zero-inflated genes

The data features that distinguish the NB distribution from ZINB are subtle, and as a result, large sample sizes are needed to identify ZI genes (Additional file 1: Fig. S2 and S4). It seems that we could avoid the problem of mis-classification by just fitting a ZINB model to each gene and reporting $\widehat {\pi }_{0}$ as a quantitative estimate of zero inflation. For example, for *Ddx3y*, after accounting for cell type, the estimated proportion of zero inflation is $\widehat {\pi }_{0}=$ 0.3326. This is comparable with *Xist* for which $\widehat {\pi }_{0}=$ 0.3518. These sex-specific genes are genuinely zero-inflated (without accounting for sex), and although they are classified differently, the $\widehat {\pi }_{0}$ values are similar. The mis-classification of *Ddx3y* is due in part to its lower overall expression level which reduces power to detect zero inflation.

**Fig. 5:**
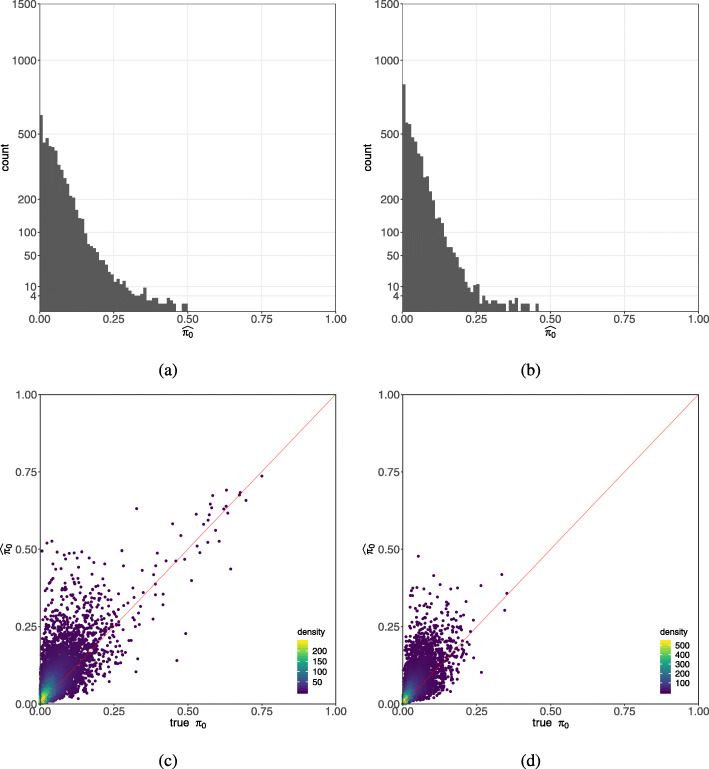
Estimating zero inflation with a ZINB model. Zero inflation probability $\widehat {\pi }_{0}$ estimated by ZINB on simulated NB data before cell type adjustment (**a**) and after cell type adjustment (**b**). Since simulated NB data does not contain zero inflation, it is implicit that the ZINB model should produce estimates of $\widehat {\pi }_{0}$ that are zero or very small. However, we find substantial overestimation of this quantity for many simulated genes. Scatterplots of true versus estimated zero inflation $\widehat {\pi }_{0}$ by ZINB on simulated ZINB data before cell type adjustment (**c**) and after cell type adjustment (**d**). Once cell type heterogeneity is regressed out, zero inflation is reduced

In order to evaluate the utility of $\widehat {\pi }_{0}$ as an indicator of zero inflation, we simulated NB and ZINB data using model parameters estimated from the heart data (*Simulation IV* in the “Methods” section). Then, we fit NB and ZINB models to each of the simulated datasets. We compared estimated values to the simulated truth (Additional file 1: Fig. S8, S9, and S10). Estimates of zero inflation from the ZINB model show a similar distribution for both the NB and ZINB simulated data (Fig. 5a, b, and Additional file 1: Fig. S10). We see that $\widehat {\pi }_{0}$ can range as high as 50% for the NB simulated data, where the true value is zero. For the ZINB simulated data, $\widehat {\pi }_{0}$ is only weakly correlated with the simulated true *π*_0_ (Fig. 5c, d). Our evaluation of $\widehat {\pi }_{0}$ suggests that it is not a reliable indicator of zero inflation.

## Discussion

Single-cell RNA sequencing data display a high frequency of zero counts. The implications of this depend on understanding the processes that give rise to zeros. Looking across the entirety of cells in an experiment, we find that a substantial number of genes meet statistical criteria for zero inflation. However, this does not necessarily imply the existence of an independent zero-generating process such as technical dropout. Instead, we find that zero inflation is largely explained by biological factors, such as cell type and sex. Recent studies of scRNA-seq in homogeneous cell populations confirm that there is no need to invoke technical dropouts as an explanation for zeros [8, 9].

A UMI count of zero does not necessarily imply that the gene is not expressed. This has led some researchers to propose imputation methods that convert zeros to non-zero values before analysis. We contend that zeros are informative data that should be incorporated directly into inferences about rates of expression and other parameters without modification. Based on the findings in this study, we and others [8, 9, 22] recommend against the practice of replacing zeros in data with imputed non-zero values as this could potentially bias estimates of gene expression, reduce signatures of stochasticity, and mask biologically relevant heterogeneity.

Model selection criteria are useful for demonstrating the presence of zero inflation, but we recommend against using a classifier to select gene-specific models for downstream analysis. This practice is known to result in inflated type I error rates, especially when the power to discriminate among models is low [23, 24]. Model averaging is one possible solution, but it can be computationally demanding and does not guarantee clear interpretation of parameters from models that we have averaged [25]. An alternative is to use a single, robust model that leads to reasonable inferences even when mis-specified. In our evaluation of the NB and ZINB models, the NB model produces accurate estimates of the mean and variance of gene expression across cells, even when applied to ZINB simulated data (Additional file 1: Fig. S8 and S9). Moreover, the NB dispersion parameter (*r*) is a good indicator of heterogeneity (Fig. 4b and Additional file 1: Fig. S11). There is no perfect model, and while the ZINB model is attractive for its generality, our simulation studies (*Simulation IV*) indicate that it may not provide reliable inferences. We recommend the generalized linear model with negative binomial errors, an offset to account for cell-to-cell variation in depth of sequencing, and including known biological factors as explanatory variables. While there are certainly opportunities to improve aspects of the negative binomial model [12, 21], it serves as the obvious default model for comparative evaluation of alternative approaches and refinements.

scRATE implements a leave-one-out cross-validation (LOO-CV) method for estimating predictive accuracy [16]. One of the appealing features of the LOO-CV approach is that it provides an estimate of the precision (the SE) of the predictive accuracy score. The SE can be used to determine when one model is significantly better than another. This is a distinct advantage compared to information criteria that provide only a score and rely on rule-of-thumb criteria to discriminate among models that are effectively equivalent [26, 27]. A drawback of the LOO-CV approach is the high computational demand. In parallel with our scRATE analysis, we computed information criterion scores. We found that AIC and BIC provided more liberal or more conservative selection, respectively, compared to scRATE, but this does not alter our main conclusion regarding the biological origins of zero inflation.

In order to understand how best to utilize the SE in model selection, we used simulated data to estimate the false negative and false positive call rates at different SE thresholds. We determined that we could use different SE thresholds based on the type of inference that we were making. To prove the existence of ZI genes, it is desirable to use a stringent threshold (2 SE or 3 SE). To generate a list of ZI genes with some tolerance for false positives, we can use a more liberal threshold (1 SE).

The scRATE software provides a powerful tool to identify ZI genes in full Bayesian context, but we do not consider it to be an essential step in standard scRNA-seq workflows. For example, the fastest way to determine that a gene is cell type-specific is to fit the NB-GLM model with and without cell type as a covariate and to compute the likelihood ratio statistic. This can be done using standard GLM software R/countreg [13]. One role for scRATE analysis would be to identify genes that, after accounting for cell type, sex, and other known covariates, still appear to be zero-inflated. In our analysis of the heart data, we identified a handful of genes, including multiple ribosomal subunit genes that are classified as ZI after accounting for cell type. The scRATE analysis draws our attention to these genes and raises open questions about the possible biological explanations for zero inflation of their count distributions.

We identified cell type as a major contributor to heterogeneity in gene expression that can explain apparent zero inflation. Data-driven clustering is not always successful in delineating cell subtypes and depends in part on the comparisons of interest to the analyst as well as the resolution with which data are viewed. Residual biological heterogeneity within a particular cell type classification may reflect distinct subgroups of cells, transient cell states, or variation along a continuum. Clustering analysis divides cells into discrete groups, but cell types are often hierarchical and distinct clusters may share different degrees of similarity [28]. Moreover, in some cases, cell “types” may exist along a continuum [29], making cluster boundaries somewhat arbitrary and dependent on features of the clustering algorithm and data. Persistence of zero inflation or high levels of overdispersion after accounting for cell type are indicators of unknown sources of biological variation that may prove to be useful in refining cell type hierarchies or positioning cells along the trajectories of a continuum.

In summary, we find substantial evidence for zero inflation in scRNA-seq data, much of which can be explained by known biological factors including cell type and sex. There remain a number of ZI genes for which we have not identified a biological explanation. Genes with zero inflation can potentially help to reveal hidden biological factors such as stage in the cell cycle, activation status of immune cells, or incomplete classification of cell types that vary across the heterogeneous mixture of cells. The model selection procedure implemented in scRATE software provides an exploratory data analysis tool for identifying these interesting genes.

## Methods

### Data

The heart data [17] consist of metabolically active, nucleated, non-myocyte cells from heart ventricles of female and male C57BL/6J mice. The dataset was sequenced on 10X Chromium scRNA-seq platform. We used the preprocessed UMI counts (downloaded from https://www.ebi.ac.uk/arrayexpress/experiments/E-MTAB-6173/), originally obtained using cellranger version 1.3 (10X Genomics). Downstream analysis using Seurat version 2.0.0 [4, 30] identified 12 cell types over 10,519 cells. In order to ensure that we include only expressed genes in our analysis, we restricted attention to 5515 genes that had at least 1 UMI in at least 10% of cells.

### Generalized linear models for count data

scRATE implements Bayesian estimation and model selection for generalized linear models (GLMs) with or without zero inflation. The distribution of counts is modeled using the log link function as a linear combination of an offset and covariates. The effect of including the offset is to account for differences in total exposure (total UMI counts per cell). With the offset, the regression parameter estimates are scaled as rates of expression in units of UMI counts per 10,000. Including a categorical covariate, e.g., cell type, allows the rates to vary across groups of cells. The zero-inflated models include a second component with zero inflation parameter *π*_0_ that represents the probability that an observed datum is an obligate zero. This component uses a logistic link function and does not require an offset. The expected number of zeros will be greater in cell types with lower rates of expression, but the proportions of extra zeros are constant across cells. Standard errors of estimated parameters are obtained by Monte Carlo sampling (scRATE) or by application of the robust sandwich estimator (CountReg [13]).

### Model selection

For counts associated with a given gene *y*_*c*_, where *c* is an index over cells, we fit Poisson, negative binomial, zero-inflated Poisson, and zero-inflated negative binomial models and evaluate their predictive accuracy based on expected log predictive density:

$$\text{ELPD}=\sum_{c=1}^{C} \log p(y_{c}|y_{-c}). $$

As a general rule, we select a model that has the largest mean ELPD as the best fit. But the data features that distinguish the best model from other models are subtle for many genes. The full Bayesian implementation provides an estimate of the mean and standard error (SE) of *ELPD difference* between models. In case two models provide similarly good fit, we select a simpler model unless the credible interval of ELPD margin offered by the other (more complex) model is always positive.

### Simulations

#### Simulation I: ZINB genes with known levels of zero inflation

We evaluated the power for detecting ZI genes by simulating ZINB data with known zero inflation probability (*π*_0_) of 10, 20, 30, …, up to 90% based on the mean and shape parameters estimated from the mouse heart data. We generated data with two different sequencing depths of 10,000 and 50,000 UMIs/cell. As sequencing depth increases, we find model under-calling substantially reduces and the proportion of correct zero inflation calls increases (Additional file 1: Fig. S2). This implies zero inflation is harder to detect when the sequencing depth is lower. We find many studies are performed below 10,000 UMIs/cell, for example, the median depth of coverage for the heart data was ∼2500 UMIs/cell.

#### Simulation II: Simulated genes with a mix of known distributions

Using model classification and parameters estimated from the heart data, we simulated P, NB, ZIP, and ZINB data for each of 5515 genes. We simulated data based on the 0 SE classification by selecting distributions for genes according to the model called at 0 SE. We repeated this process using model calls at 1 SE, 2 SE, and 3 SE. We applied scRATE model selection with the 0, 1, 2, and 3 SE thresholds to each simulated gene set. These model calls allow us to compute true and false positive rates for detecting ZI genes and to compute the AUC for each combination of simulation and evaluation thresholds (Additional file 1: Fig. S3). We find that the 1 SE threshold provides the best balance between false positive (over-calling of model) and false negative (under-calling of model) classification.

#### Simulation III: Random subsets of cells

The number of cells may have a substantial impact on the power of detecting ZI genes. In order to assess the effect of cell number on detecting ZI genes, we generated random subsets of cells by down-sampling data from the 10,519 cells in the mouse heart data. The number of cells ranges from 44 up to 9000 (Additional file 1: Fig. S4). These random subsets retain the heterogeneity of original data, and therefore, the number of ZI genes should not appreciably change with the number of cells, except due to loss of power for detecting ZI genes. We found that the number of ZI genes increases with the number of cells sampled (Additional file 1: Fig. S4). This implies the number of ZI genes we detected in the original dataset should be regarded as a lower bound.

#### Simulation IV: NB versus ZINB

To evaluate the effect of naïvely applying a ZINB model to non-zero-inflated data, we simulated 5515 genes from an NB distribution with parameters estimated from the heart data. We fit both NB and ZINB models to simulated NB data and evaluated the parameter estimates including $\widehat {\pi }_{0}$ for each gene. We find that fitting NB data with the ZINB model yielded high estimates of zero inflation for many genes. Next, we simulated 5515 genes from a ZINB distribution with parameters estimated from the heart data. We fit both NB and ZINB models to the simulated ZINB data. ZINB is the correct model in this simulation, but we find that $\widehat {\pi }_{0}$ estimation is still unstable for many genes (Fig. 5c, d) although overall it has lower mean square errors than NB (Additional file 1: Table S2). We also find that NB leads to reasonable inferences even when mis-specified in this simulation.

## Supplementary information

**Additional file 1** Application of scRATE method to additional datasets, followed by Supplementary Figures and Tables.

**Additional file 2** Review history.
