# Publisher Correction: Bayesian model selection reveals biological origins of zero inflation in single-cell transcriptomics

**DOI:** 10.1186/s13059-020-02182-1

**Published:** 2020-11-03

**Authors:** Kwangbom Choi, Yang Chen, Daniel A. Skelly, Gary A. Churchill

**Affiliations:** 1 The Jackson Laboratory, 600 Main Street, Bar Harbor, ME 04609 USA; 2 University of Michigan, 500 South State Street, Ann Arbor, MI 48109 USA

**Correction to: Genome Biol (2020) 21:183**

**https://doi.org/10.1186/s13059-020-02103-2**

Following publication of the original paper [1], it was identified that the Background section of this article was missing from the HTML version. The original article [1] has been corrected.
