# Supplementary material for: Bayesian model selection reveals biological origins of zero inflation in single-cell transcriptomics
Source: Genome Biol. 2020 Jul 27;21:183. doi: 10.1186/s13059-020-02103-2 (PMC7384222; doi:10.1186/s13059-020-02103-2)
Supplement: Supplementary file 1 — Additional file 1 Application of scRATE method to additional datasets, followed by Supplementary Figures and Tables. [file 13059_2020_2103_MOESM1_ESM.pdf]

**Supplementary Materials: Bayesian model selection reveals biological origins of zero inflation in single-cell transcriptomics**

### *Additional Dataset I: the mouse kidney data*

This dataset [1] is a single cell transcriptome atlas of mouse kidney. The library was prepared with the whole kidney tissues of seven healthy male C57BL/6 mice using 10X Chromium (v2 chemistry) protocol and sequenced on Illumina HiSeq 2000 platform. We downloaded a processed data file available at NCBI's GEO under accession number GSE107575. It includes 43,745 cells consisting of 16 distinct cell types: endothelial, vascular, descending loop of Henle, podocyte, proximal tubule, ascending loop of Henle, distal convoluted tubule, collecting duct (CD) principal cell, CD intercalated cell, CD transitional cell, fibroblast, macrophage, neutrophil, natural killer cell in addition to two novel cell types. In order to ensure that we include only expressed genes in our analysis, we restricted attention to 5,160 genes that had at least 1 UMI in at least 10% of cells, the same filtering criterion used for the heart data.

### *Additional Dataset II: the human PBMC data*

This dataset [2] is peripheral blood mononuclear cells (PBMCs) from a healthy donor, sequenced on Illumina NovaSeq platform with ∼54,000 reads per cell. We downloaded a processed data file [3] available at `https://www.dropbox.com/s/zn6khirjafoyyxl/pbmc_10k_v3.rds?dl=0`. It contains 9,432 cells consisting of 14 cell types: CD14+ Monocytes, CD4 Memory, CD4 Naive, pre-B cell, Double negative T cell, NK cell (bright and dim), B cell progenitor, CD8 effector, CD8 Naive, CD16+ Monocytes, Dendritic cell, pDC, Platelet. The raw data are also available at `https://support.10xgenomics.com/single-cell-gene-expression/datasets/3.0.0/pbmc_10k_v3`. In order to ensure that we include only expressed genes in our analysis, we restricted attention to 6,435 genes that had at least 1 UMI in at least 10% of cells, the same filtering criterion used for the heart data.

**References**


[1] Park J, Shrestha R, Qiu C, Kondo A, Huang S, Werth M, et al. Single-cell transcriptomics of the mouse kidney reveals potential cellular targets of kidney disease. Science. 2018;360(6390):758–763. Available from: https://science.sciencemag.org/content/360/6390/758.

[2] 10X Genomics. 10k PBMCs from a Healthy Donor (v3 chemistry); 2018. Accessed: Oct. 13th, 2019. https://support.10xgenomics.com/single-cell-gene-expression/datasets/3.0.0/pbmc_10k_v3.

[3] Stuart T, Butler A, Hoffman P, Hafemeister C, Papalexi E, Mauck WM, et al. Comprehensive Integration of Single-Cell Data. Cell. 2019;177(7):1888–1902.e21. Available from: http://www.sciencedirect.com/science/article/pii/S0092867419305598.

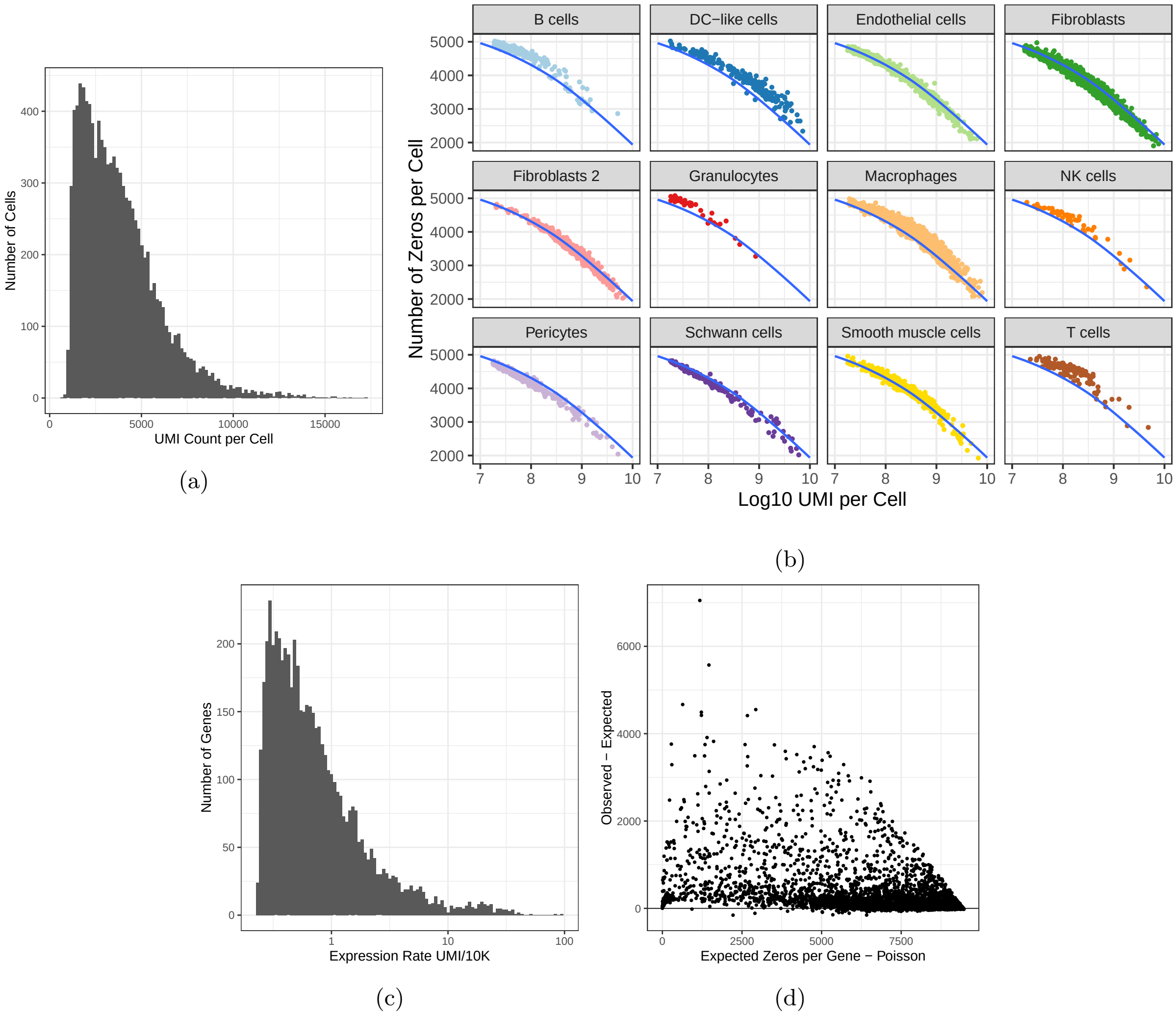


Fig. S1: Factors that determine the number of zeros in scRNA-Seq data. (a) Total UMI counts per cell, which range from 746 to 17,302 with average 3,819 UMIs per cell, are shown as a histogram. (b) The number zeros per cell is plotted against the log10 UMI count. The plot is facetted to show the individual cell types as determined by data-driven clustering. The blue line shows the loess fit to the combined data. (c) The per-gene rates of expression ($\mu_g$), which range from 0.23 to 97.4 with average 1.51 UMI/10K, are shown as a histogram. (d) A scatter plot shows the expected number of zeros under Poisson sampling (x-axis) compared to the difference from expectation (observed - expected; y-axis) and reveals genes with an excess of zeros.

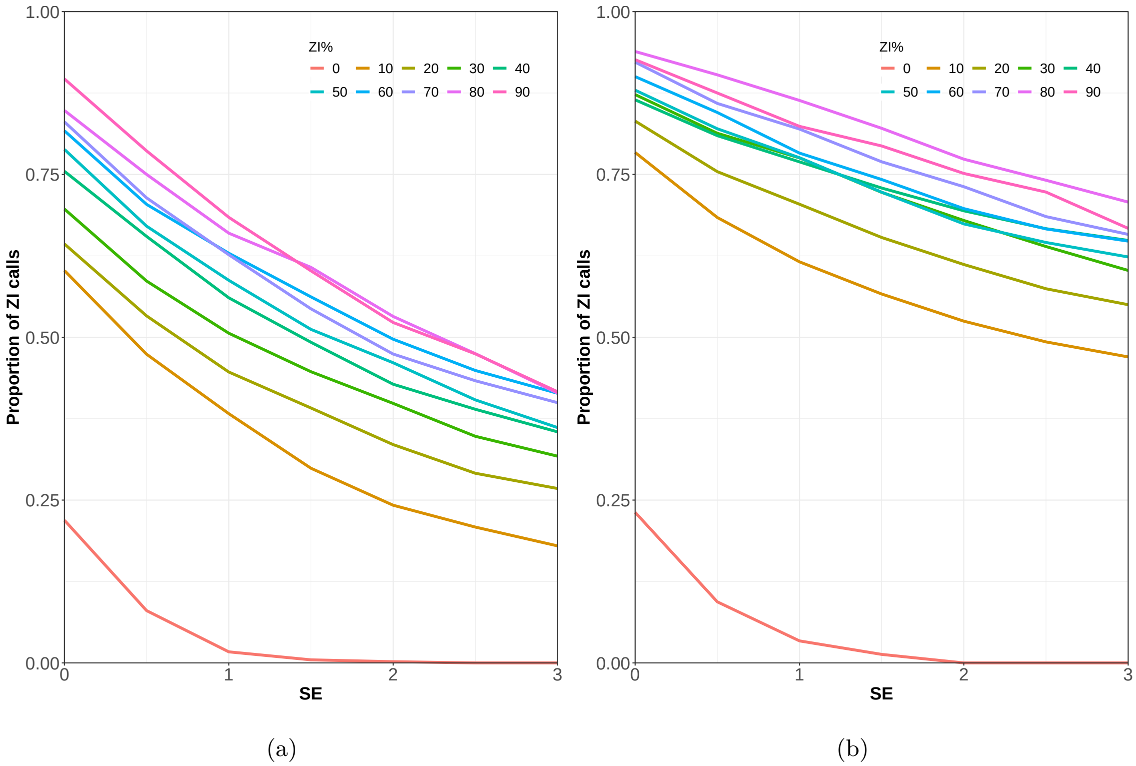


Fig. S2: Power analysis of `scRATE` classification of ZI gene. We simulated data as described in Methods (*Simulation I*) across a range of zero inflation (0 to 90%, color coded). We applied `scRATE` with thresholds ranging from 0 SE to 3 SE (x-axis) and counted the proportion of genes classified and ZIP or ZINB (y-axis). The red line shows the numbers of ZI genes detected when the simulation model is NB, with no zero inflation. This is the false positive rate, or type-I error, of the classifier. The other lines indicate different proportion of zero inflation in the ZINB model. This is true positive rate, or power, of the classifier. Simulations are based on sequence depths of 10,000 UMIs (a) and 50,000 UMIs per cell (b).

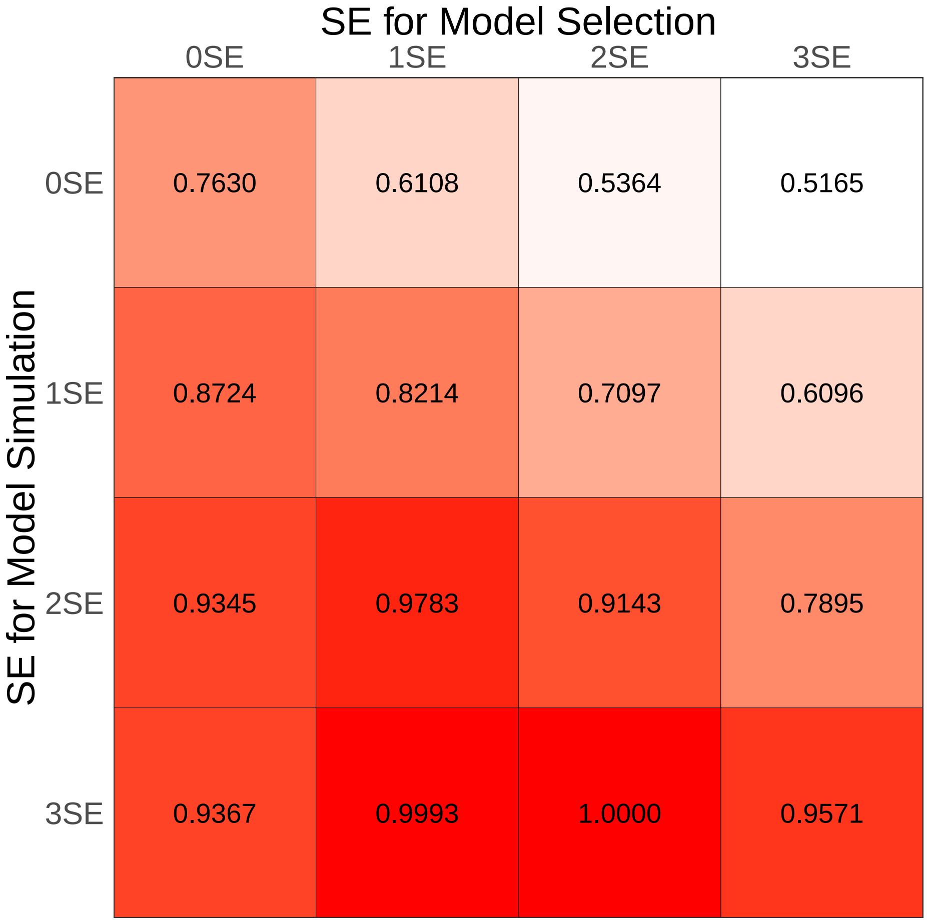


Fig. S3: Area Under ROC Curve (AUC) of different model selection thresholds. As shown in Fig. S2, there is a trade off along the stringency of threshold: the more stringent it gets, the rate of model under-calling (false negative rate) increases while the rate of over-calling (false positive rate) reduces. In order to identify the optimal threshold, we evaluated AUC with simulated gene sets (*Simulation II*) for which distributions are selected from the heart data with the 0, 1, 2, and 3 SE thresholds (rows). For each simulated gene set, we performed `scRATE` classification with the 0, 1, 2, and 3 SE thresholds (columns). We find that the 1 SE threshold (the second column) is robust and performs relatively well across the simulated gene sets. See Fig. S13 and S14 for the results with the mouse kidney and the human PBMC data sets.

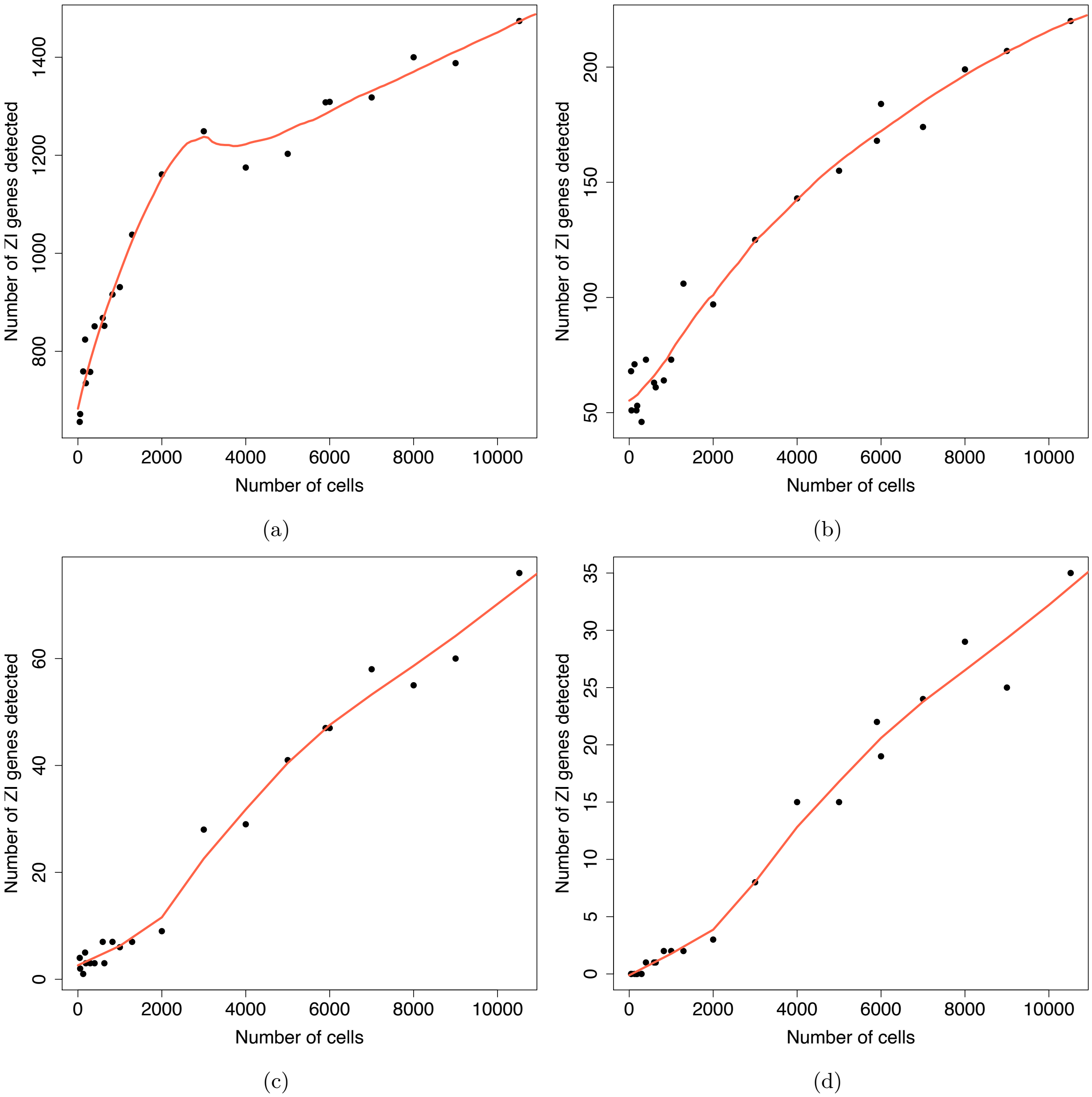


Fig. S4: Detection of ZI genes in down-sampled heart data. We randomly sampled subset of cells (*Simulation III*) across a range of sample sizes (x-axis), applied `scRATE` to the reduced data, and counted the numbers of ZI genes (y-axis) at the threshold of 0 SE (a), 1 SE (b), 2 SE (c), and 3 SE (d). Red line is a lowess fit.

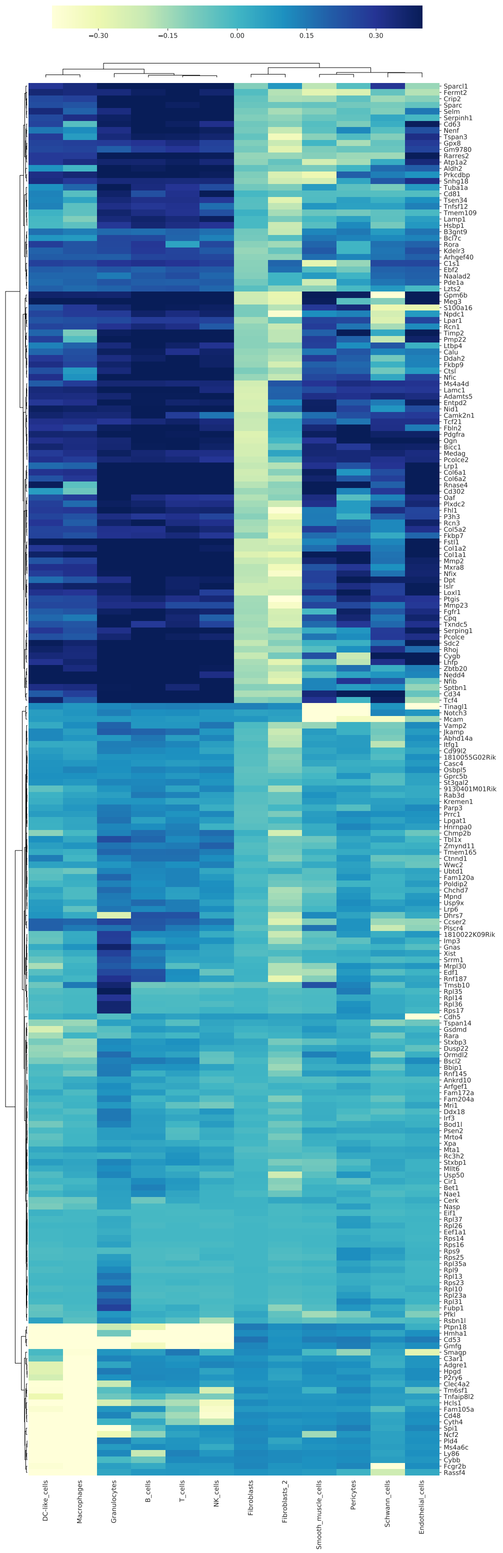

Fig. S5: Zeros cluster within specific cell types for ZI genes in the heart data. A bi-clustered heatmap of ZI genes (1 SE) by cell types shows the deviation from expected number of cells with zero UMI counts. Dark shading indicates an excess of zeros and light shading indicates that the cell type has fewer cells with zero UMI count than expected. See Tables S15 and S16 for the results with the mouse kidney and the human PBMC data sets.

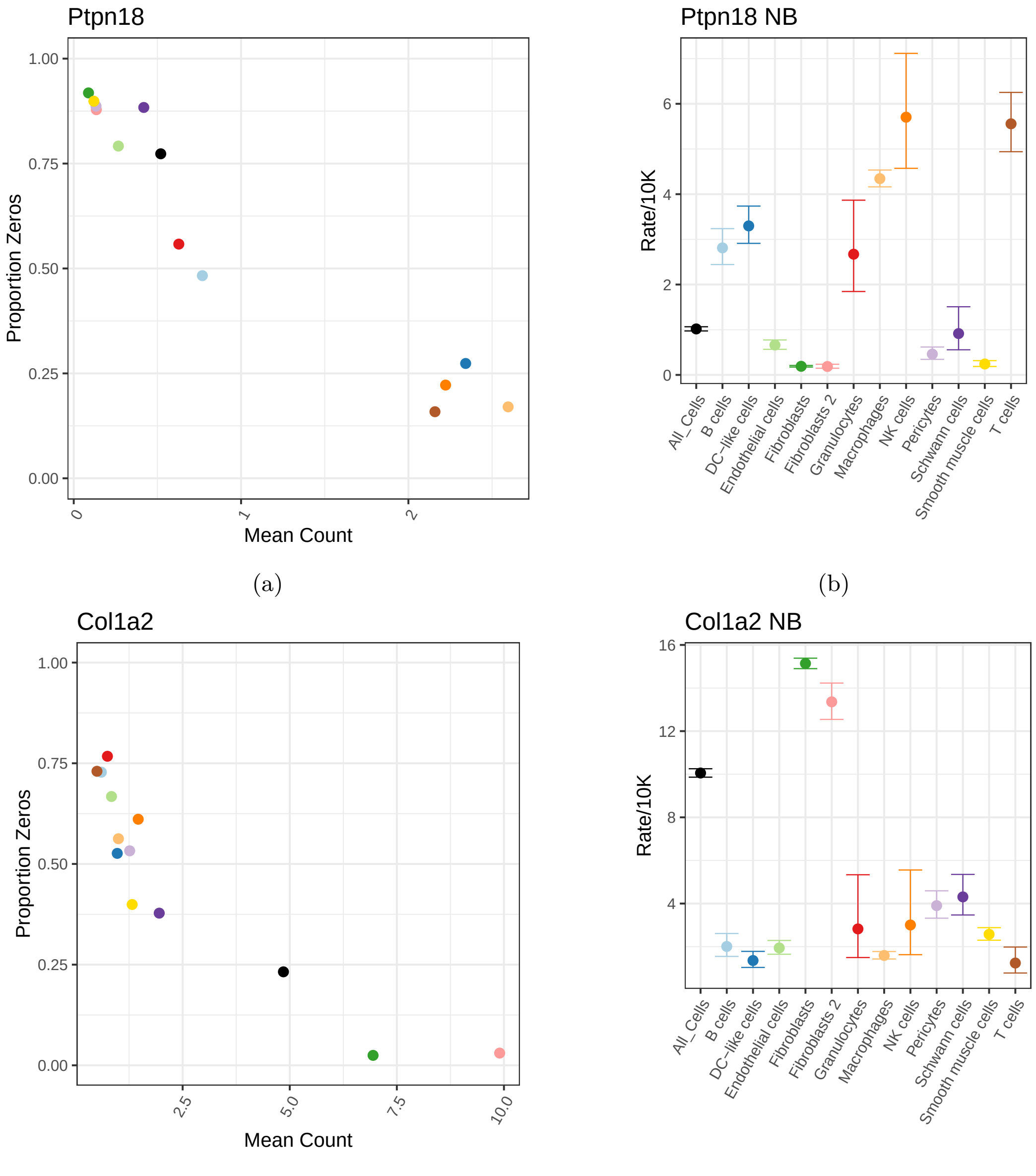


(c) (d)

Fig. S6: Examples of ZI genes that are no longer ZI after accounting for cell type. *Ptpn18* is primarily expressed in immune cells. The upper left panel shows the proportion of zeros, averaged across cells within each cell type, as a function of the mean UMI count per cell. The upper right panel shows the estimated rates of expressed for NB model overall (All Cells) and for each cell type as estimated by `scRATE` with cell type as covariate. Lower panels show the same for *Col1a2*, a gene primarily expressed in fibroblasts.

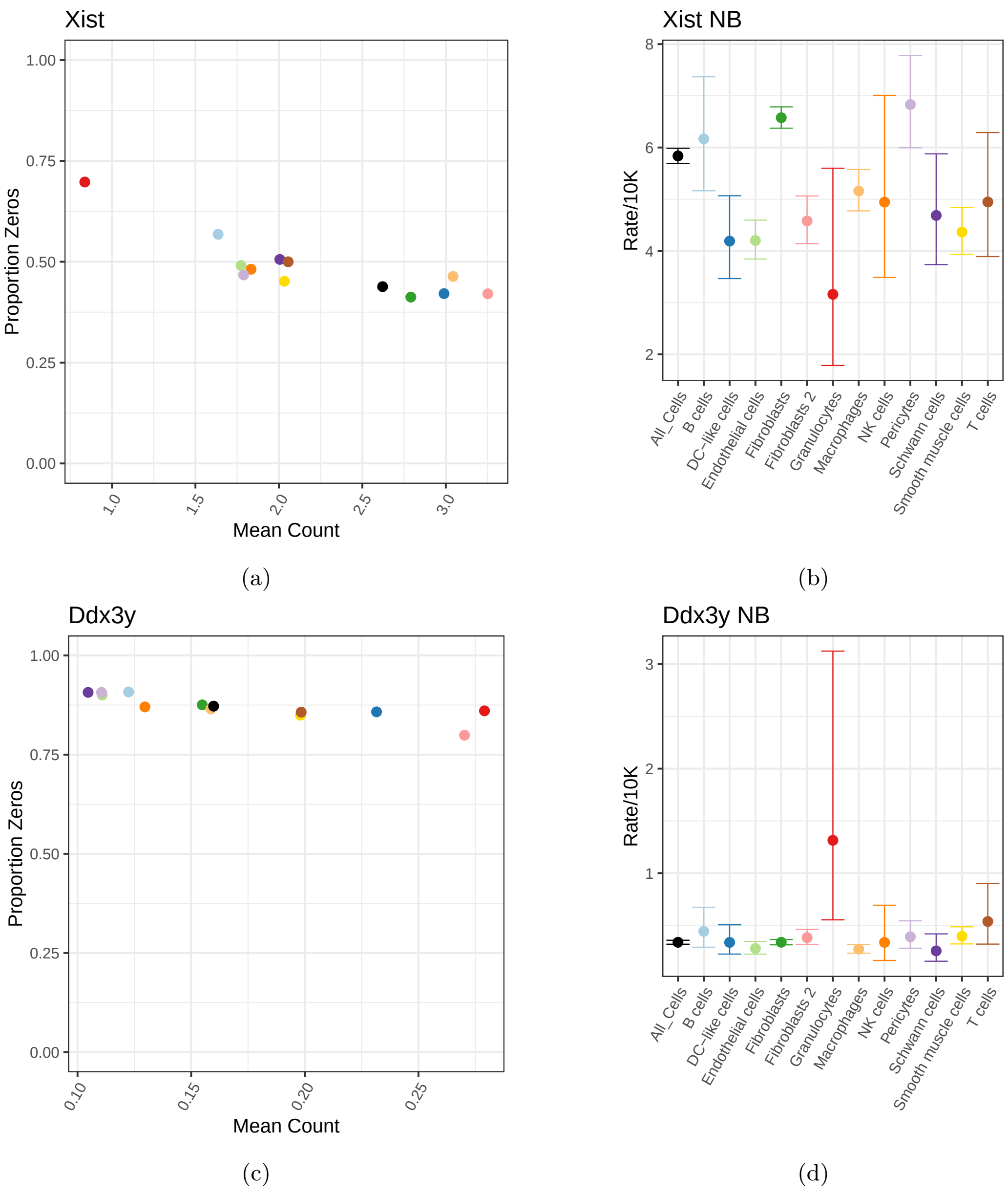


Fig. S7: Examples of ZI genes that remain ZI after accounting for cell type. *Xist* is a female specific transcript encoded on the X chromosome. *Ddx3y* is a male specific gene encoded on the Y chromosome. Panels are as described for Fig. S6. There appears to be a high proportion of granulocytes among the male cells.

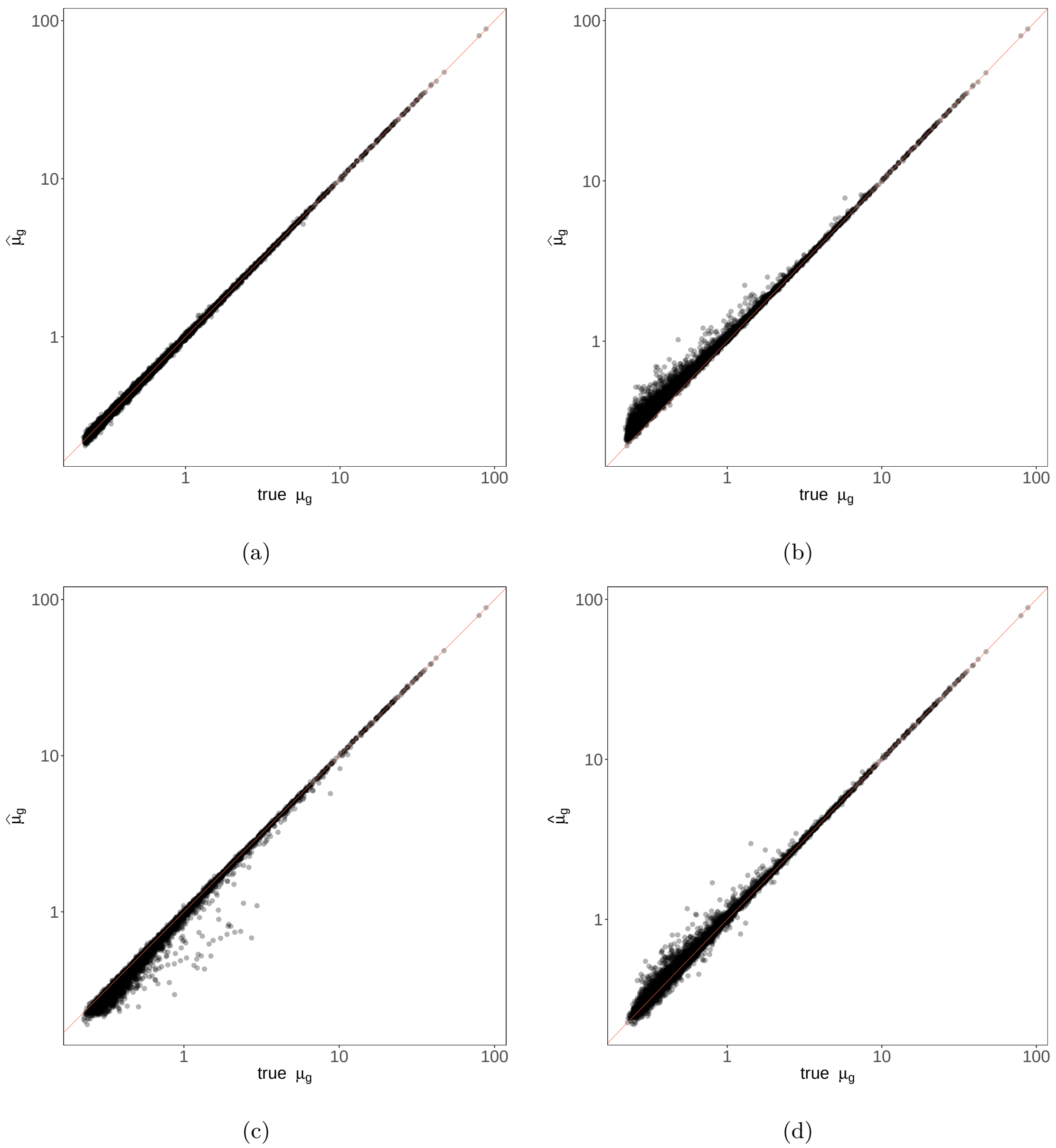


Fig. S8: Estimation of the mean (rate of expression) parameter in simulated data. Data were simulated under either the NB model or ZINB model as described in Methods (*Simulation IV*). All panels show estimated rates (y-axis) are compared to simulation truth (x-axis). (a) Fitting NB model to NB simulated data. (b) Fitting ZINB model to NB simulated data. (c) Fitting NB model to ZINB simulated data. (d) Fitting ZINB model to ZINB simulated data. The biases observed in (b) and (c) are explained by the different interpretation of mean rate between the NB and ZINB models.

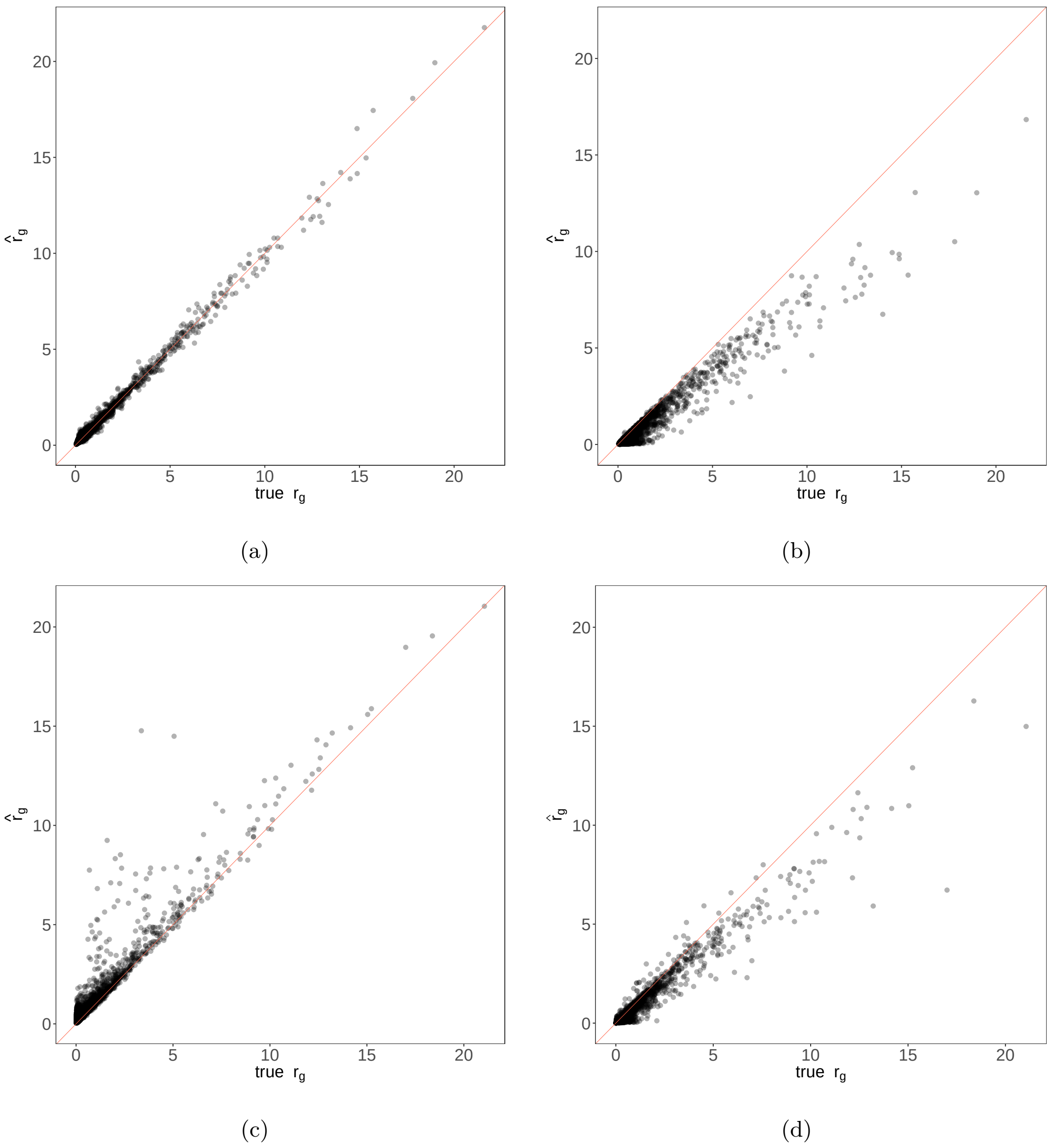


Fig. S9: Estimation of the overdispersion in simulated data. Data were simulated under either the NB model or ZINB model as described in Methods (*Simulation IV*). All panels show estimated overdispersion (y-axis) are compared to simulation truth (x-axis). (a) Fitting NB model to NB simulated data. (b) Fitting ZINB model to NB simulated data. (c) Fitting NB model to ZINB simulated data. (d) Fitting ZINB model to ZINB simulated data. The biases observed in (b) and (c) illustrate how the overdispersion and zero inflation parameters trade-off, with one compensating for the other when fitted model is mis-specified relative to the data.

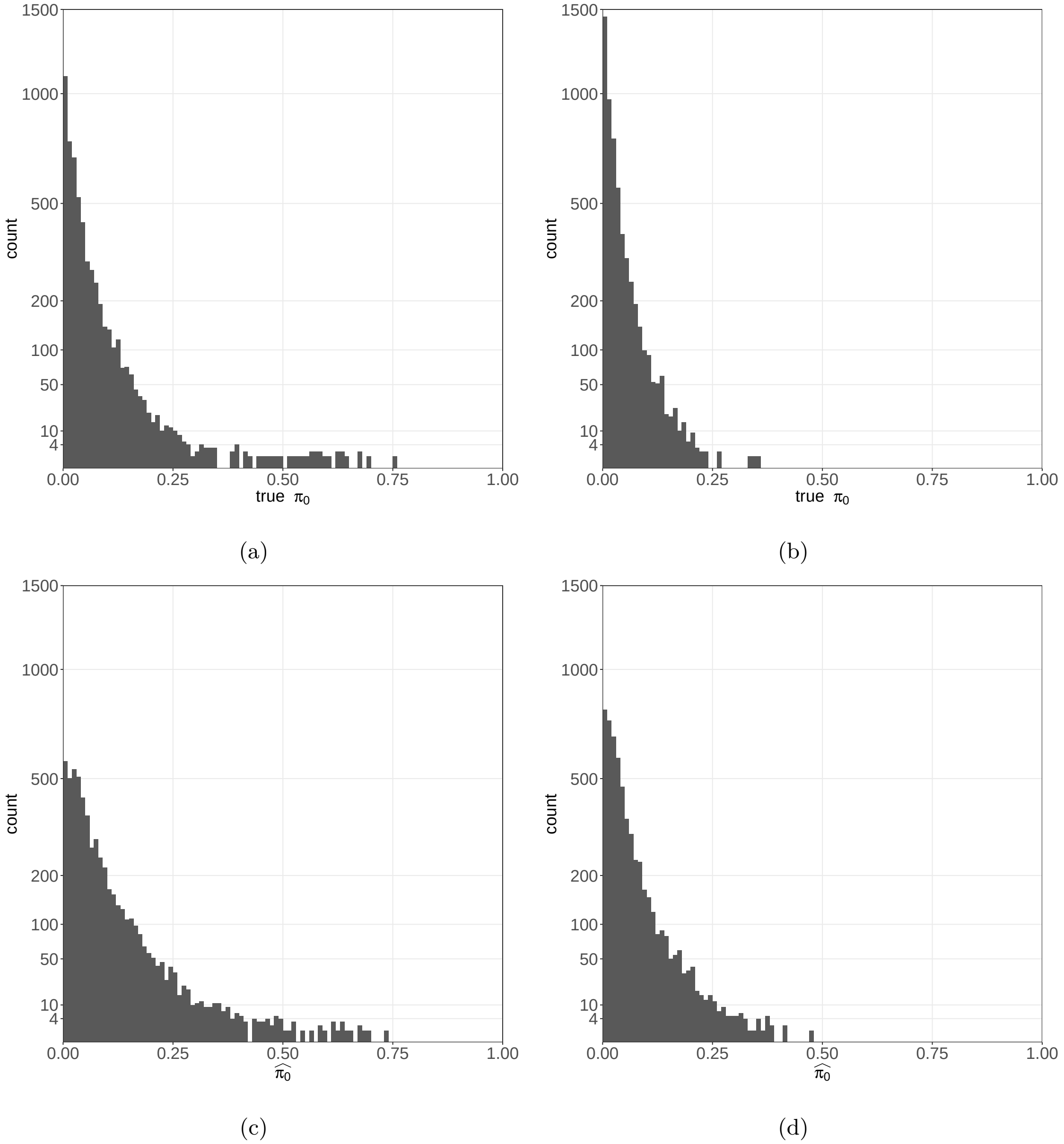


Fig. S10: Estimated zero inflation on simulated ZINB data. True zero inflation $\pi_0$ estimated from the mouse heart data before cell type adjustment (a) and after cell type adjustment (b). Estimated zero inflation $\widehat{\pi}_0$ on simulated ZINB data before cell type adjustment (c) and after cell type adjustment (d). Cell type adjustment reduces the estimated amount of zero inflation overall. The variability of estimated zero inflation in simulation increases (c)(d) compared to that of real data (a)(b).

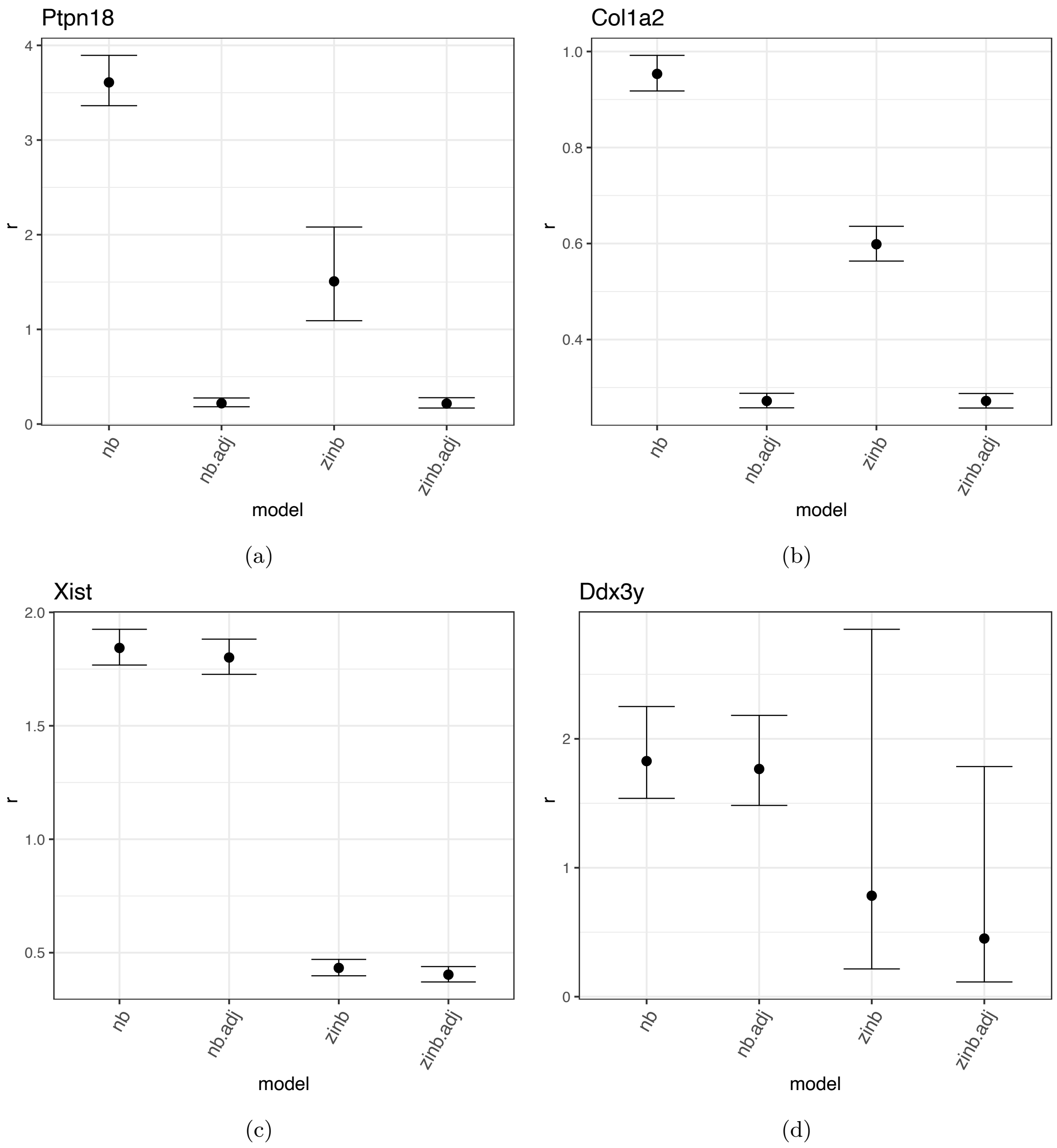


Fig. S11: Effects of cell type and zero inflation on estimated overdispersion. The overdispersion parameter (y-axis) was estimated using the NB model, with and without cell type as a covariate and again using the ZINB model with and without cell type. For the cell type-specific genes *Ptpn18* and *Col1a2*, there is a small reduction in overdispersion from the NB to ZINB model without cell type. Including cell type as a covariate reduces overdispersion to almost zero. For the sex-specific genes *Xist* and *Ddx3y*, the inclusion of cell type has negligible effect on overdispersion whereas allowing for zero inflation reduces overdispersion substantially.

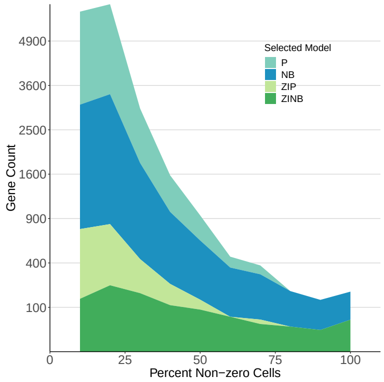


(a)

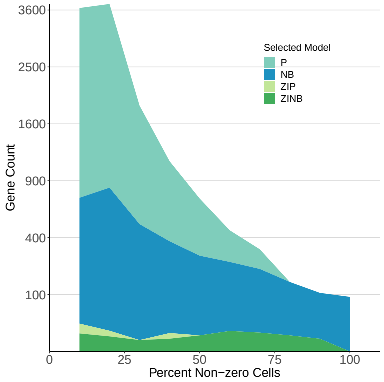


(b)

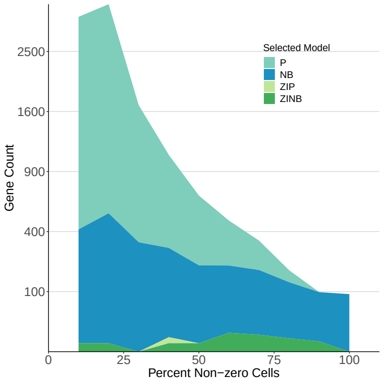


(c)

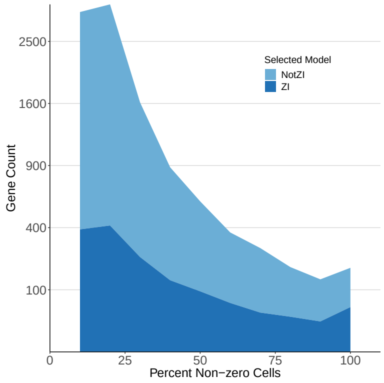


(d)

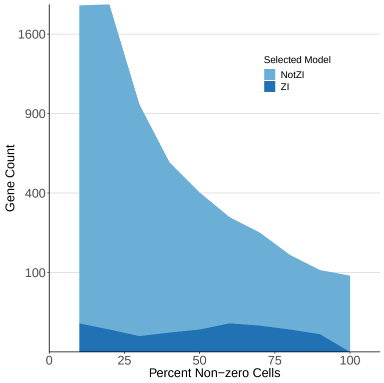


(e)

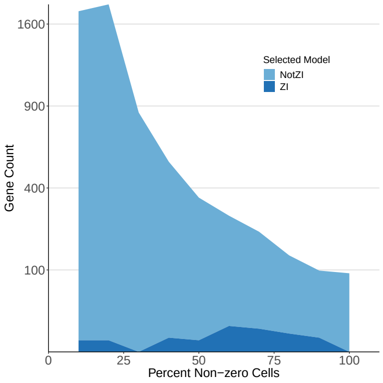


(f)

Fig. S12: Classification of genes by `scRATE` model selection applied to heart data. (a) Density plot of model classification of genes across percentages of non-zero cells using `scRATE` with the 0 SE threshold. (b) As above, for the 2 SE threshold. (c) As above, for the 3 SE threshold. Density plots of `scRATE` classification collapsed to show only the ZI versus NotZI genes across percentages of non-zero cells with the 0 SE (d), 2 SE (e), and 3 SE (f) thresholds as indicated. See Fig. S17 and S18 for the results with the mouse kidney and the human PBMC data sets.

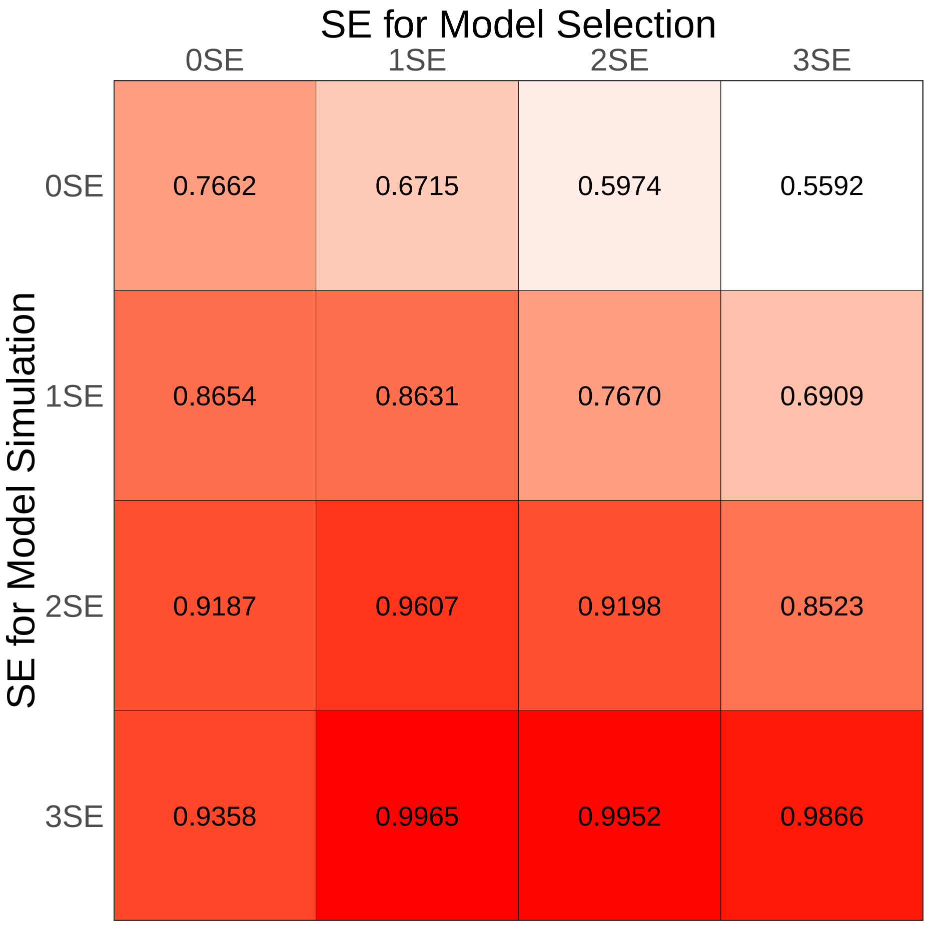


Fig. S13: AUC of different model selection thresholds with the simulation based on the kidney data. In order to identify the optimal threshold as in Fig. S3, we evaluated AUC with simulated gene sets for which distributions are selected from the kidney data with the 0, 1, 2, and 3 SE thresholds (rows). For each simulated gene set, we performed `scRATE` classification with the 0, 1, 2, and 3 SE thresholds (columns). We find that the 1 SE threshold (the second column) is again robust and performs relatively well across the simulated gene sets.

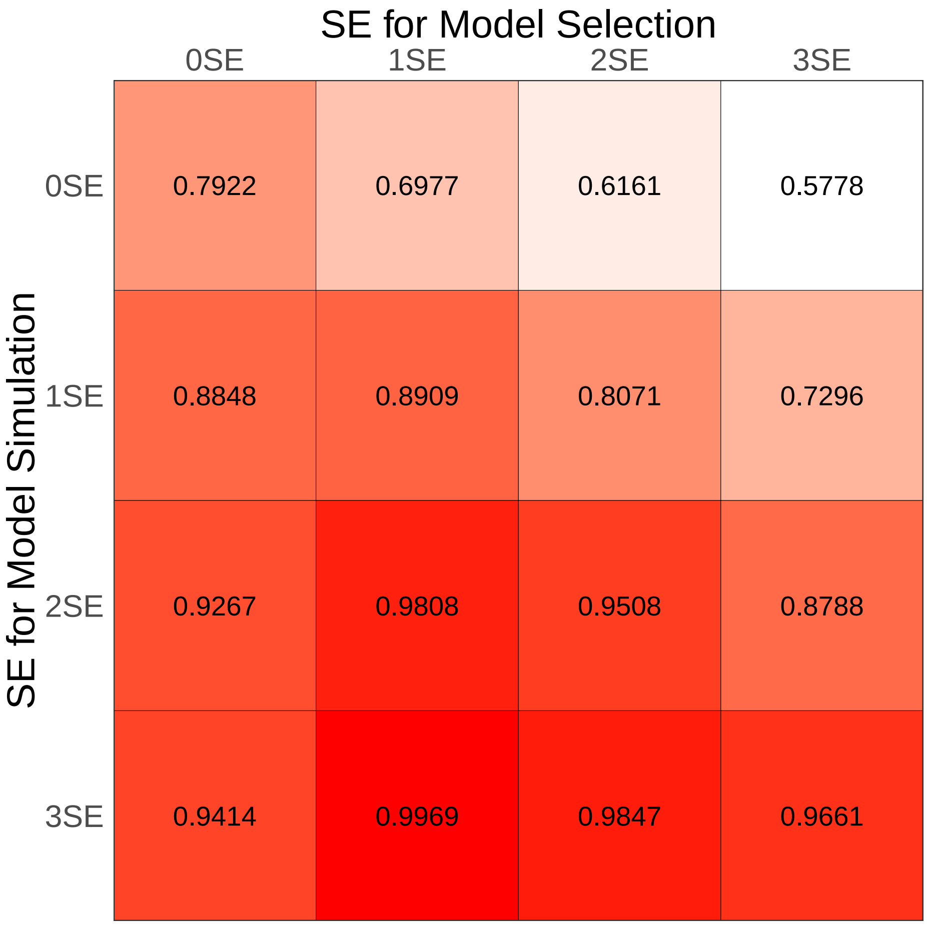


Fig. S14: AUC of different model selection thresholds with the simulation based on the PBMC data. In order to identify the optimal threshold as in Fig. S3 and S13, we evaluated AUC with simulated gene sets for which distributions are selected from the PBMC data with the 0, 1, 2, and 3 SE thresholds (rows). For each simulated gene set, we performed `scRATE` classification with the 0, 1, 2, and 3 SE thresholds (columns). We find again that the 1 SE threshold (the second column) is robust and performs relatively well across the simulated gene sets.

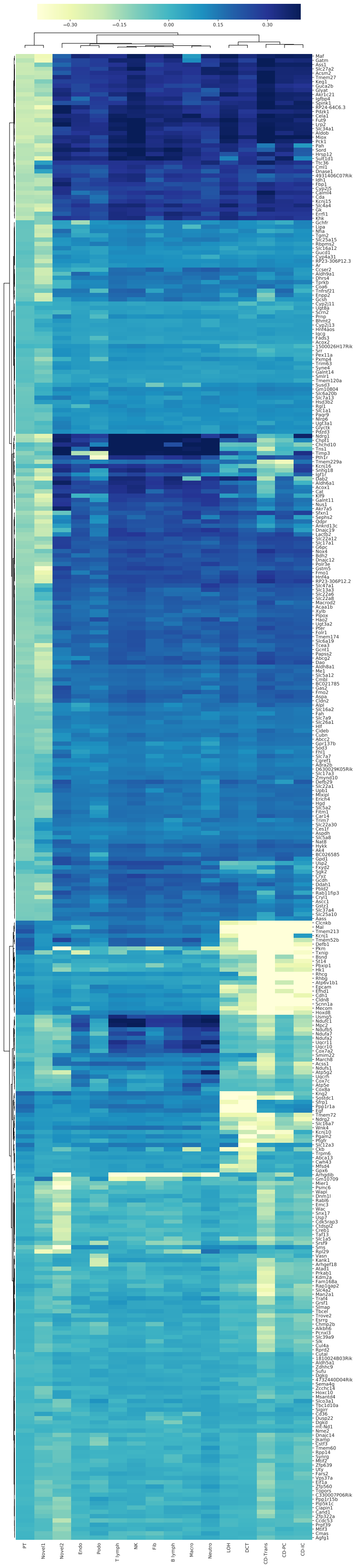


Fig. S15: Zeros cluster within specific cell types for ZI genes in the kidney data. A bi-clustered heatmap of ZI genes (1 SE) by cell types shows the deviation from expected number of cells with zero UMI counts. Dark shading indicates an excess of zeros and light shading indicates that the cell type has fewer cells with zero UMI count than expected.

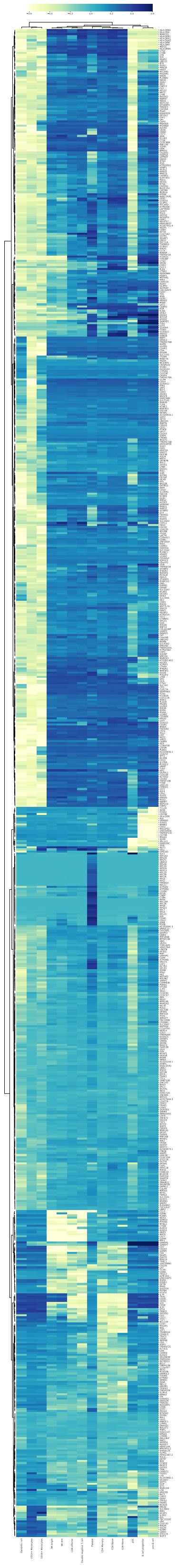

Fig. S16: Zeros cluster within specific cell types for ZI genes in the PBMC data. A bi-clustered heatmap of ZI genes (1 SE) by cell types shows the deviation from expected number of cells with zero UMI counts. Dark shading indicates an excess of zeros and light shading indicates that the cell type has fewer cells with zero UMI count than expected.

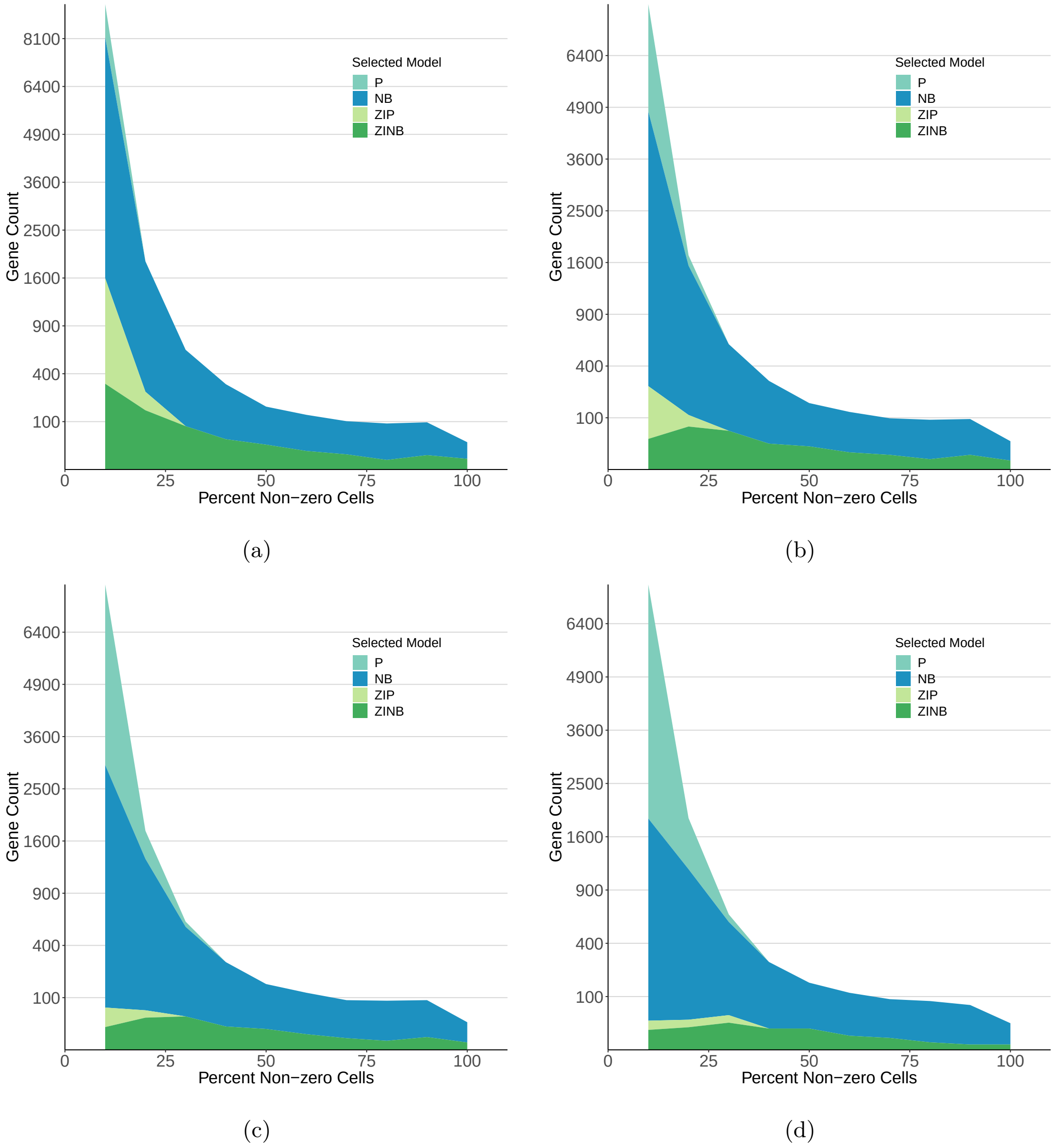


Fig. S17: Classification of genes by `scRATE` model selection applied to the kidney data. (a) Density plot of model classification of genes across percentages of non-zero cells using `scRATE` with the 0 SE threshold. (b) As above, for the 1 SE threshold, (c) for the 2 SE threshold, and (d) for the 3 SE threshold.

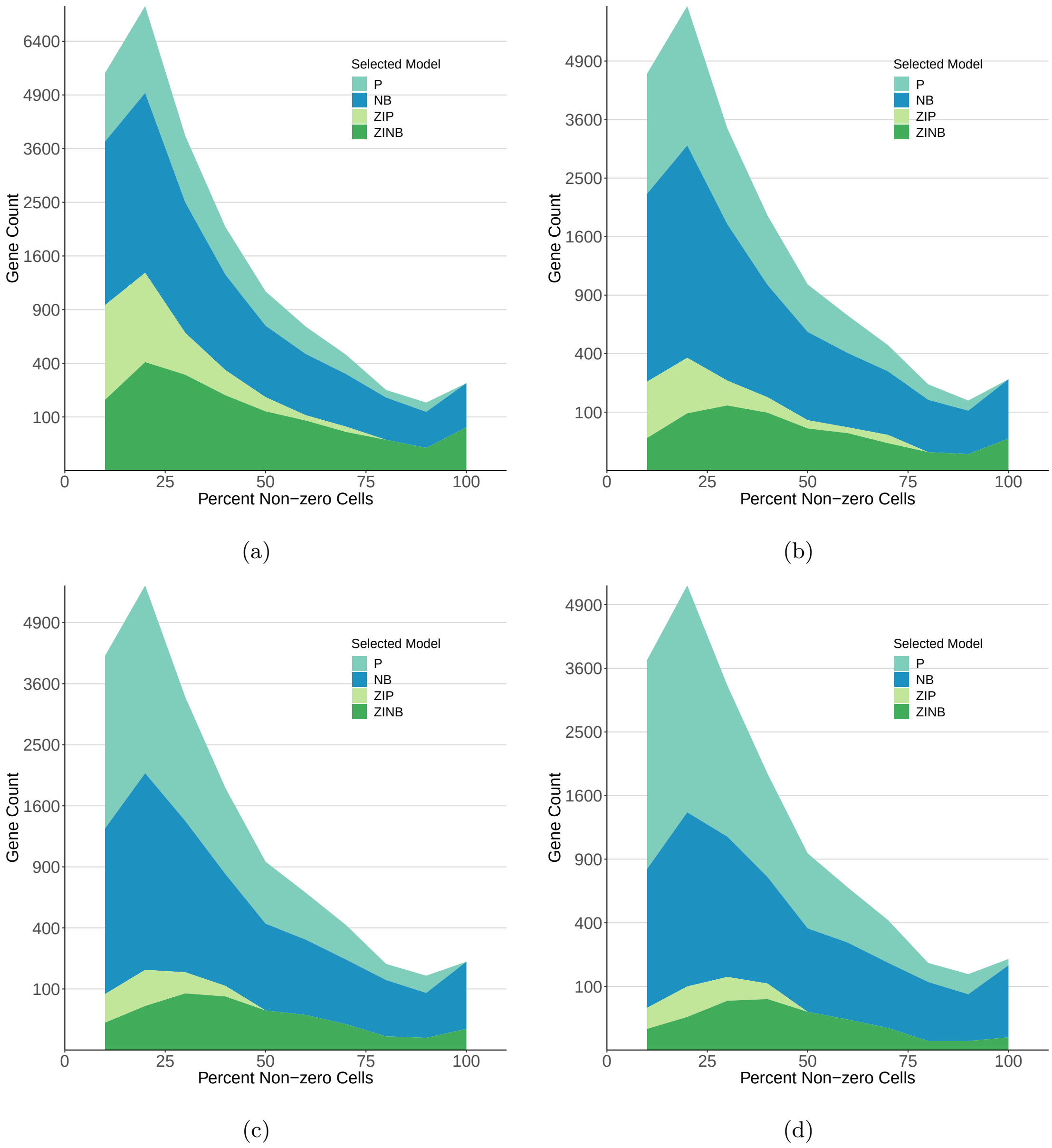


Fig. S18: Classification of genes by `scRATE` model selection applied to the PBMC data. (a) Density plot of model classification of genes across percentages of non-zero cells using `scRATE` with the 0 SE threshold. (b) As above, for the 1 SE threshold, (c) for the 2 SE threshold, and (d) for the 3 SE threshold.

Table S1: Properties of genes classified by `scRATE`

(a)

| 0 SE model | NumBest | mNumZeros | medNumZeros | mPctNZ | medPctNZ | medAvgExpr | mAvgExpr |
|---|---|---|---|---|---|---|---|
| P | 1,111 | 8,366 | 8,710 | 20.4 | 17.1 | 0.192 | 0.251 |
| NB | 2,930 | 7,384 | 8,159 | 29.7 | 22.4 | 0.299 | 0.756 |
| ZIP | 525 | 8,581 | 8,865 | 18.3 | 15.6 | 0.178 | 0.223 |
| ZINB | 949 | 6,467 | 7,293 | 38.5 | 30.6 | 0.415 | 1.27 |

(b)

| 1 SE model | NumBest | mNumZeros | medNumZeros | mPctNZ | medPctNZ | medAvgExpr | mAvgExpr |
|---|---|---|---|---|---|---|---|
| P | 2,112 | 8,340 | 8,694 | 20.6 | 17.3 | 0.196 | 0.257 |
| NB | 3,183 | 7,119 | 7,924 | 32.3 | 24.6 | 0.338 | 0.889 |
| ZIP | 81 | 8,216 | 8,465 | 21.8 | 19.4 | 0.229 | 0.287 |
| ZINB | 139 | 4,553 | 4,435 | 56.7 | 57.8 | 1.39 | 3.05 |

(c)

| 2 SE model | NumBest | mNumZeros | medNumZeros | mPctNZ | medPctNZ | medAvgExpr | mAvgExpr |
|---|---|---|---|---|---|---|---|
| P | 2,930 | 8,285 | 8,676 | 21.2 | 17.4 | 0.200 | 0.268 |
| NB | 2,509 | 6,733 | 7,612 | 35.9 | 27.6 | 0.412 | 1.16 |
| ZIP | 5 | 8,546 | 9,127 | 18.7 | 13.1 | 0.148 | 0.249 |
| ZINB | 71 | 5,074 | 4,455 | 51.7 | 57.6 | 1.41 | 1.89 |

Table S2: Mean square errors of mean and overdispersion parameters for the genes in the heart data (a) NB simulation before cell type adjustment, (b) ZINB simulation before cell type adjustment, (c) NB simulation using cell type as covariate, and (d) ZINB simulation using cell type as covariate.

(a)

| NB simulation | NB | ZINB |
|---|---|---|
| mean | 0.00153 | 0.00811 |
| overdispersion | 0.0166 | 0.2817 |

(b)

| ZINB simulation | NB | ZINB |
|---|---|---|
| mean | 0.01385 | 0.00587 |
| overdispersion | 0.24941 | 0.14867 |

(c)

| NB simulation | NB | ZINB |
|---|---|---|
| mean | 0.00548 | 0.00777 |
| overdispersion | 0.009 | 0.052 |

(d)

| ZINB simulation | NB | ZINB |
|---|---|---|
| mean | 0.00812 | 0.00705 |
| overdispersion | 0.0444 | 0.0165 |

Table S3: Number of models called for the genes in the kidney data without using cell type as covariate (a), using cell type as covariate (b), and using cell type as covariate after randomly shuffling cell type labels (c).

(a)

| Threshold | Selected model | | | |
|---|---|---|---|---|
| | P | NB | ZIP | ZINB |
| 0 SE | 48 | 3,946 | 500 | 666 |
| 1 SE | 434 | 4,378 | 109 | 239 |
| 2 SE | 1,225 | 3,760 | 16 | 159 |
| 3 SE | 2,024 | 3,023 | 7 | 106 |

(b)

| Threshold | Selected model | | | |
|---|---|---|---|---|
| | P | NB | ZIP | ZINB |
| 0 SE | 104 | 3,654 | 485 | 917 |
| 1 SE | 730 | 4,221 | 117 | 92 |
| 2 SE | 1,807 | 3,311 | 9 | 33 |
| 3 SE | 2,679 | 2,471 | 0 | 10 |

(c)

| Threshold | Selected model | | | |
|---|---|---|---|---|
| | P | NB | ZIP | ZINB |
| 0 SE | 52 | 3,820 | 459 | 829 |
| 1 SE | 447 | 4,367 | 111 | 235 |
| 2 SE | 1,272 | 3,711 | 18 | 159 |
| 3 SE | 2,053 | 2,995 | 7 | 105 |

Table S4: The number of models called for the genes in the PBMC data without using cell type as covariate (a), using cell type as covariate (b), and using cell type as covariate after randomly shuffling cell type labels (c).

(a)

| Threshold | Selected model | | | |
|---|---|---|---|---|
| | P | NB | ZIP | ZINB |
| 0 SE | 743 | 3,536 | 680 | 1,476 |
| 1 SE | 1,536 | 4,174 | 213 | 512 |
| 2 SE | 2,566 | 3,448 | 72 | 349 |
| 3 SE | 3,458 | 2,682 | 54 | 241 |

(b)

| Threshold | Selected model | | | |
|---|---|---|---|---|
| | P | NB | ZIP | ZINB |
| 0 SE | 1,307 | 2,941 | 701 | 1,486 |
| 1 SE | 2,574 | 3,572 | 142 | 147 |
| 2 SE | 3,930 | 2,460 | 6 | 39 |
| 3 SE | 4,833 | 1,588 | 1 | 13 |

(c)

| Threshold | Selected model | | | |
|---|---|---|---|---|
| | P | NB | ZIP | ZINB |
| 0 SE | 752 | 3,383 | 639 | 1,661 |
| 1 SE | 1,556 | 4,147 | 221 | 511 |
| 2 SE | 2,630 | 3,383 | 76 | 346 |
| 3 SE | 3,486 | 2,652 | 55 | 242 |
