# Supplementary material for: Bayesian model selection reveals biological origins of zero inflation in single-cell transcriptomics
Source: Genome Biol. 2020 Jul 27;21:183. doi: 10.1186/s13059-020-02103-2 (PMC7384222; doi:10.1186/s13059-020-02103-2)
Supplement: Supplementary file 2 — Additional file 2 Review history. [file 13059_2020_2103_MOESM2_ESM.docx]

Review History

**First round of review**

**Reviewer 1**

**Are you able to assess all statistics in the manuscript, including the appropriateness of statistical tests used?**

Yes, and I have assessed the statistics in my report.

**Comments to author:**

In this manuscript Choi et al present a Bayesian approach for analyzing zero inflation in scRNA-seq data. The large numbers of zeros in scRNA-seq data is a salient feature and there has been considerable work in the field seeking to quantify, understand and deal with these dropouts. As the debate is still ongoing, this manuscript provides a welcome contribution. The paper uses a novel model fitting approach to compare models with and without ZI to conclude that ZI can to a large extent be explained by known biological factors. Overall, I find the paper well-written and easy to follow. The methodology seems sound, and the conclusions are well motivated.

I have the following major comments:

1 In the introduction it is mentioned that one should expect to observe the Poisson distribution due to equal proportions. I am not sure that I agree with this assumption, if one assumes that the proportion is a random variable instead (which is reasonable given all the fluctuations), then long tailed distributions emerge more readily. I think that this ought to be clarified in the text.

2 The authors rightly point out that it is important to consider covariates when determining whether or not a distribution is zero-inflated. Unsurprisingly, they identify the sequencing depth as the variable which explains most of the zeros. A very useful dataset for this purpose is the one provided by Bacher et al (https://www.nature.com/articles/nmeth.4263) which includes the same cell type sequenced at two different depths. I would like the authors to apply their model to this dataset to learn how well it generalizes.

3 What is the justification for using the leave-one-out procedure to account for model complexity? How can one best intuitively understand the use of different SE thresholds? Would methods such as AIC or BIC give the same result?

4 Although I was able to install the scRATE package, there was no documentation available. This state of affairs is not acceptable and I am unwilling to recommend a paper for publication without this basic requirement fulfilled.

5 Looking at fig S6, I do not agree with the authors' assertion that saturation is approached for 10k genes in a and b. I agree that the curve is starting to taper off, but in both cases it is hard to see what the asymptote should be. The authors need to either revise or strengthen this argument.

6 Based on the results in Fig 2, the authors argue that ZI genes are not more lowly expressed. However, as far as I can tell, the simulation protocol does not control for cell type. As expression level and ZI are both functions of cell type, I would think that one must control for this aspect (as is done later). Could the authors explain why this feature was not considered here?

7 Intuitively, it is easy to understand why there is ZI (transcripts are lost during the sample prep). However, it is more difficult to understand why there should be fewer zeros than expected (as is frequently reported in fig 3). Could the authors please comment on what they believe is the source of this?

8 To test the validity and generality of the authors' model, I would like to see it applied to at least one more potential explanatory variable. For example, cell cycle is often highlighted as such a variable and I would like to know how well the method works for this variable (eg https://www.biorxiv.org/content/10.1101/526848v5). Alternatively, could the model be applied to a differentiation time-course ('pseudotime') to show that one could use such a feature as an explanatory factor?

I have the following minor comments:

1 Could the authors run their simulations for Table 1 multiple times to provide error bars to the reported estimates?

2 Figure 3 is very frustrating for an old curmudgeon with poor vision such as this reviewer. If the intention is that readers should be able to make out the cell type and gene names they need to be made larger. Otherwise, I think that it is better to leave them out. Alternatively, if there are biologically meaningful groups of genes identified, then it would be useful to highlight these.

3 For fig 4a, could the authors add an arrow to point out Ddx3y?

**Reviewer 2**

**Are you able to assess all statistics in the manuscript, including the appropriateness of statistical tests used?**

Yes, and I have assessed the statistics in my report.

**Comments to author:**

The manuscript "Bayesian model selection reveals biological origins of zero inflation in single-cell transcriptomics" explores zero inflation and describes a method for identifying genes that are zero inflated. The authors find that zero inflation is highly dependent on cell type and sex. Overall, the message of the paper is that imputation is likely unnecessary (at best) and instead zeros should be considered part of the expression distribution. The R package described may be useful in exploring zero-inflated genes for a given dataset. This paper brings together some conflicting views on analyzing single cell transcriptomic data and provides conclusions that are clear and well supported. This paper will be of wide interest.

Specific comments:

1. It is not clear how this statement was calculated and I didn't see it in the methods: "Sequencing depth explains 95% of variation in the number of zeros per cell."

2. The authors should cite this additional paper that discusses comparing models for UMI data:

"UMI-count modeling and differential expression analysis for single-cell RNA sequencing"

Wenan Chen, Yan Li, John Easton, David Finkelstein, Gang Wu & Xiang Chen

3. The package and its general use are not thoroughly described. The authors might discuss how one could identify genes that are cell-type specific in their expression based on whether it is zero-inflated (i.e. a gene is ZI when ignoring cell type and is no longer ZI after accounting for it because it's expressed in only one subset of cells.), or other ideas along those lines.

4. The package currently has no vignette on how to run the different functions. I was not able to test it out on my own.

**Authors Response**

**Point-by-point responses to the reviewers’ comments:**

Reviewer #1: In this manuscript Choi et al present a Bayesian approach for analyzing zero

inflation in scRNA-seq data. The large numbers of zeros in scRNA-seq data is a salient feature and there has been considerable work in the field seeking to quantify, understand and deal with these dropouts. As the debate is still ongoing, this manuscript provides a welcome contribution. The paper uses a novel model fitting approach to compare models with and without ZI to conclude that ZI can to a large extent be explained by known biological factors. Overall, I find the paper well-written and easy to follow. The methodology seems sound, and the conclusions are well motivated.

I have the following major comments:

1 In the introduction it is mentioned that one should expect to observe the Poisson distribution due to equal proportions. I am not sure that I agree with this assumption, if one assumes that the proportion is a random variable instead (which is reasonable given all the fluctuations), then long tailed distributions emerge more readily. I think that this ought to be clarified in the text.

*If the mRNA proportions are equal and the only variation in counts is due to statistical sampling, the distribution will be Poisson. However, we agree with the reviewer that these assumptions that give rise to the Poisson distribution are unlikely to apply to real data. We made this statement simply to introduce the Poisson distribution as the default model for count data. In practice, we recommend the use of an over-dispersed model such as the negative binomial distribution. For clarity we have modified the text as follows:*

*“If the frequency of a given mRNA species is uniform across cells and variation from cell to cell is due only to independent statistical sampling, counts would follow a Poisson distribution. However, the assumptions that give rise to the Poisson distribution are unrealistic due to numerous biological and technical factors that will cause mRNA to vary from cell to cell. As a result, count data will be more variable than predicted by a Poisson distribution. The negative binomial distribution provides a good approximation for the distribution of UMI count data (Chen et al., 2018). It assumes that the underlying mRNA frequencies are random and the excess variability of observed counts is quantified by an overdispersion parameter, r.”*

2 The authors rightly point out that it is important to consider covariates when determining whether or not a distribution is zero-inflated. Unsurprisingly, they identify the sequencing depth as the variable which explains most of the zeros. A very useful dataset for this purpose is the one provided by Bacher et al (https://www.nature.com/articles/nmeth.4263) which includes the same cell type sequenced at two different depths. I would like the authors to apply their model to this dataset to learn how well it generalizes.

*We appreciate the reviewer pointing out the work by Bacher. Their data are based on the automated microfluidic platform (Fluidigm C1), but the experimental design can be used to directly observe the effect of read depth on the count distribution. We note that total counts in Bacher are quite high: 0.3 to 2.1M reads/cell at low coverage and 1.2 to 9.2M reads per cell at high coverage in comparison to depths of 5~25k UMIs in typical droplet-based scRNA-seq data sets. As a result, the proportion of zeros per cell is substantially smaller than our heart data or other droplet-based scRNA-Seq data that we have examined.*

*Nonetheless we can confirm that the number of zero is determined by read depth (Figure R1). The proportion of variation in number of zeros explained by read depth (R2=0.384) is lower than for the heart data.*

*Figure R1: The relationship between sequencing depth and the number of zeros in scRNA-seq data using microfluidic platform (Fluidigm C1). Total UMI counts per cell are plotted against the number of zeros per cell. The number of zeros is largely determined by read depth (R2=0.384).*

*The number of cells surveyed in the Bacher experiments is small (92 cells per experiment) and there is little or no cell type heterogeneity by design. Accordingly, we applied scRATE with an offset term but with no other covariates. In the low read-depth experiments, we identified 607 and 229 ZI genes and in the high read-depth experiments, we identified 273 and 295 ZI genes, using 1SE criterion.*

*The Bacher experiment was carried using cells of the same type. However, when the cells are clustered based on the zeros in ZI genes, we see evidence of some degree of cell heterogeneity (Figure R2).*

*Figure R2: The heatmap of gene expression level for ZI genes in Bacher et al. 2015 data*

*To avoid lengthy digression, we have added a brief comment and reference in the discussion:*

*“While our focus here is on droplet-based scRNA-Seq which yields low total UMI counts on very large numbers of cells, scRATE can be applied to data obtained by single-cell sequencing methods that produce deeper coverage of smaller numbers of cells (e.g., Bacher et al., 2015).”*

*Also because the use of offsets is not familiar to readers who may not have formal training in the generalized linear model, we clarified it in the subsection “Why are there so many zeros?”: “To account for variation in sequencing depth we incorporate log of the total UMI count per cell as an offset in the generalized linear model. An offset can be thought of as a covariate for which the regression parameter is fixed and not estimated. Hafemeister and Satija (2019) have recently shown that treating the offset as a covariate with an estimated coefficient leads to overfitting. The effect of including an offset is simply to rescale the data from a count to a rate. In addition to the overfitting problem, the use of a fixed regression coefficient provides a consistent interpretation of the estimated model parameters as a rate of expression. For example, if we set the offset to log(total UMI count per cell /10e5), the mean parameters from any of the GLMs (P, NB, ZIP or ZINB) can be interpreted as the expected gene-specific UMI count per 10,000 total UMI.”*

3 What is the justification for using the leave-one-out procedure to account for model complexity? How can one best intuitively understand the use of different SE thresholds? Would methods such as AIC or BIC give the same result?

*“All models are wrong, but some are useful.” When evaluating the utility of a model, we can evaluate how well the model can predict outcomes that were not included in the model estimation (training), i.e., the predictive accuracy of the models. Our choice to implement leave-one-out cross validation (LOO-CV) in scRATE was based on work of Vehtari et al (2017) who demonstrate superior performance of LOO-CV on estimating predictive accuracy of models compared to information criteria including AIC, BIC and WAIC. Although the LOO-CV procedure is computationally demanding, it appealed to us because it provides an estimate of the precision (the SE) of the predictive accuracy score. The SE associated with a score can be used to determine when the one model is significantly better than another.*

*Information criteria including AIC and BIC produce a score but rely ‘rule-of-thumb’ criteria to decide when to distinguish among models with similar scores. We used simulated data to estimate the false negative and false positive rates of scRATE at different SE thresholds. We recognized that we could use different SE thresholds to control the two error rates depending on the type of inference we were making. To prove the existence of ZI genes, it was desirable to use a stringent threshold (2SE or 3SE). To generate lists of ZI genes with tolerance for false positives we used a more liberal threshold (1SE).*

*In parallel with the scRATE analysis, we computed all of the model comparisons in this study using AIC and BIC. We found that AIC is generally more liberal and BIC is more conservative. However, evaluating model selection criteria was not our objective and describing these comparisons became a distraction from the main point of the paper – which is that biological factors can explain zero inflation in scRNA-Seq data. These information criteria lead to same conclusion regarding the causes of zero inflation.*

*We added the following text to the discussion in order clarify these points:*

*scRATE implements a leave-one-out cross validation (LOO-CV) method for estimating predictive accuracy (Vahari et al., 2017). One of the appealing features of the LOO-CV approach is that it provides an estimate of the precision (the SE) of the predictive accuracy score. The SE can be used to determine when one model is significantly better than another. This is a distinct advantage compared to information criteria that provide only a score and rely on rule-of-thumb criteria to discriminate among models that are effectively equivalent (Gelman et al., 2014; Dziak et al., 2019). A drawback of the LOO-CV approach is the high computational demand. In parallel with our scRATE analysis, we computed information criterion scores. We found that AIC and BIC provided more liberal or more conservative selection, respectively, compared to scRATE but this does not alter our main conclusion regarding the biological origins of zero inflation.*

*In order to understand how best to utilize the SE in model selection, we used simulated data to estimate the false negative and false positive call rates at different SE thresholds. We determined that we could use different SE thresholds based on the type of inference that we were making. To prove the existence of ZI genes, it is desirable to use a stringent threshold (2SE or 3SE). To generate a list of ZI genes with some tolerance for false positives we can use a more liberal threshold (1SE).*

4 Although I was able to install the scRATE package, there was no documentation available. This state of affairs is not acceptable and I am unwilling to recommend a paper for publication without this basic requirement fulfilled.

*We sincerely regret this oversight. The package is now provided with installation instructions, documentation of the scRATE functions and sample code on the package GitHub page. The containers in which scRATE is preinstalled are also available at Docker and Singularity hubs. In addition, we have revised the Software section in Methods.*

*Software Availability*

*Documentation and sample use cases for scRATE are available on our GitHub repository (https://github.com/churchill-lab/scRATE). To run scRATE, first prepare a dataframe with the UMI counts of a gene, the cell-specific offsets, and covariates. The offsets are log total UMI count for each cell scaled as desired, e.g., use log(total count/10^5) to obtain mean parameters scaled to UMI per ten-thousand. Covariates should be categorical (factors) or, if continuous, are assumed to have a linear trend effect on the mean rate of expression. The model fitting, leave-one-out cross validation, and model selection steps are executed one gene at a time using scRATE functions model_fit(), compare_count_models(), and select_model(), respectively. Due to the computational demand local computing applications should be limited to a few genes. We have containerized the software (using both docker (https://hub.docker.com/r/kbchoi/scrate) and singularity (https://singularity-hub.org/collections/4398)) to facilitate its use on high-performance computing clusters or with cloud computing.*

5 Looking at fig S6, I do not agree with the authors' assertion that saturation is approached for 10k genes in a and b. I agree that the curve is starting to taper off, but in both cases it is hard to see what the asymptote should be. The authors need to either revise or strengthen this argument.

*We agree with reviewer’s assessment and have revised text (on p. 8) as follows:*

*The number of ZI genes detected continues to increase with the number of cells even up to 10,000 cells. This suggests that the number of ZI genes detected, especially at the stringent 2SE threshold, is an underestimate of the actual number of ZI genes.*

6 Based on the results in Fig 2, the authors argue that ZI genes are not more lowly expressed. However, as far as I can tell, the simulation protocol does not control for cell type. As expression level and ZI are both functions of cell type, I would think that one must control for this aspect (as is done later). Could the authors explain why this feature was not considered here?

*Figure 2 is based on a real dataset (mouse heart). Our intention was to illustrate how the data would be interpreted without consideration of cell type as has been done in previous studies. In particular Karchenko et al., 2014, which appears to be the first paper to make specific reference to “dropouts”, argues that the number of extra zeros due to technical artifact increases as the expression level of a gene decreases. In Figure 2, we show that zero inflation is detected in higher expressed genes as well. We have looked at the expression levels of ZI genes selected both with and without cell type as a covariate. We find the ZI genes that remain after accounting for cell type are even more highly expressed. This implies that zero inflation is due to the presence of cell type(s) in which those zero-inflated genes are not expressed but are expressed, sometimes at high levels in other cell types. It can be difficult to distinguish zero inflation from non-zero inflated sampling zeros in cases where cell types that express a gene do so at low levels. To clarify this point, we included the following (new text underlined):*

*However, our findings, appear to support the opposite conclusion ... The statistical test for zero inflation will have best power in cases where the gene is not expressed in some cell type(s) but, when it is expressed, expression levels are high. When expression levels are low across all cell types, zero-inflation is hard to distinguish from statistical sampling from a non-zero-inflated distribution. As a result, genes that are declared to be ZI using statistical testing are potentiailly biased toward higher levels of expression. Nonetheless, the number of zeros alone ...*

7 Intuitively, it is easy to understand why there is ZI (transcripts are lost during the sample prep). However, it is more difficult to understand why there should be fewer zeros than expected (as is frequently reported in fig 3). Could the authors please comment on what they believe is the source of this?

*This is a misunderstanding that suggests we need to revise the figure caption. Our intention is to show that zeros are not distributed evenly across cell types for the ZI genes. For each gene g, let "# be the proportion of cells of type c with zero counts. Let ̅# be the mean proportion of zeros across all cells without regard to cell type. For some cell types, the cell-type specific proportion "# must be greater than the overall mean ̅# and for others it must be less. The display is showing deviations of the cell-type specific proportions from their overall mean "#– ̅#. We found this visualization to be most effective to display data across genes with a wide range of average rates of expression. The updated figure caption is:*

*Figure 3: A bi-clustered heatmap of ZI genes (1 SE) by cell type shows that zeros occur more frequently in specific cell types. The color scale indicates the difference between the cell-type specific proportion of zeros and the mean proportion of zeros across all cells regardless of type. Light shading indicates cell types that have highest frequency of zero UMI counts.*

8 To test the validity and generality of the authors' model, I would like to see it applied to at least one more potential explanatory variable. For example, cell cycle is often highlighted as such a variable and I would like to know how well the method works for this variable (eg https://www.biorxiv.org/content/10.1101/526848v5). Alternatively, could the model be applied to a differentiation time-course ('pseudotime') to show that one could use such a feature as an explanatory factor?

*After accounting for cell type and sex, a handful of genes in the heart data are still classified as ZI genes. The genes appear to be enriched in functional classes, e.g. there are a substantial number of ribosomal genes that remain in the ZI group. We speculated that other biological factors could account for these ZI genes if they were somehow known. Cell cycle is an obvious possibility. However, we were not able to demonstrate a cell cycle effect in the heart data. As suggested by the reviewer, we examined data from Hsiao et al. 2020 which identified 520 cell cycle genes. None of them were zero inflated at the stringency level of 2 SE. But at 1 SE, one of four ZINB genes is no longer zero inflated when cell cycle is used as a covariate. When we lower the stringency down to 0 SE, more genes (22 of 82 ZINB genes) are not called as zero inflated after cell cycle as a covariate (Table R1). Our treatment of cell cycle as a categorical variable is not ideal. Hsiao et al. model cell cycle as a as continuous variable (ranging from 0 to 2π) and changes in gene expression are cyclic. scRATE is limited to modeling categorical variables or continuous variables with a linear trend and extending it to model the sinusoidal model of cell cycle gene expression does not seem feasible at this time.*

*We have included this analysis as a vignette to accompany the scRATE software but we have not incorporated it into the revised manuscript.*

I have the following minor comments:

1 Could the authors run their simulations for Table 1 multiple times to provide error bars to the reported estimates?

*Good suggestion. The revised version of Table 1 now includes standard errors based on replicated simulation runs.*

2 Figure 3 is very frustrating for an old curmudgeon with poor vision such as this reviewer. If the intention is that readers should be able to make out the cell type and gene names they need to be made larger. Otherwise, I think that it is better to leave them out. Alternatively, if there are biologically meaningful groups of genes identified, then it would be useful to highlight these.

*As described in response to comment 7, our intention is to visualize uneven distribution of zeros among cell subtypes. We felt that it was important to see the entire heat map on one page which causes a problem for labeling the y-axis. We have created an alternative figure that breaks the heat map spanning four columns and provide a new Figure 3. We note that the original figure is a high-resolution image that can be expanded on a computer monitor for easier viewing.*

3 For fig 4a, could the authors add an arrow to point out Ddx3y?

*Revised figure with labeling of Ddx3y, Xist and additional genes of interest.*

Reviewer #2: The manuscript "Bayesian model selection reveals biological origins of zero inflation in single-cell transcriptomics" explores zero inflation and describes a method for identifying genes that are zero inflated. The authors find that zero inflation is highly dependent on cell type and sex. Overall, the message of the paper is that imputation is likely unnecessary (at best) and instead zeros should be considered part of the expression distribution. The R package described may be useful in exploring zero-inflated genes for a given dataset. This paper brings together some conflicting views on analyzing single cell transcriptomic data and provides conclusions that are clear and well supported. This paper will be of wide interest.

Specific comments:

1 It is not clear how this statement was calculated and I didn't see it in the methods: "Sequencing depth explains 95% of variation in the number of zeros per cell."

*This is the R2 value after fitting a loess curve to the data as shown in the figure. The most convenient place to clarify this point was in the figure caption.*

*Old Text: Sequencing depth explains 95% of variation in the number of zeros per cell (R2 = 0.945, p < 2.2e-16). We account for this variation by including an offset term in the GLM, which we have scaled to represent rates of expression in units of UMI/10k.*

*New Text: To account for variation in sequencing depth we incorporate log of the total UMI count per cell as an offset in the generalized linear model (Methods).*

*New text in figure caption: The proportion of variance in the number of zeros that is explained by the total UMI count per cell (R2=0.947) was computed based on fitting a loess regression to the data (blue curve).*

2 The authors should cite this additional paper that discusses comparing models for UMI data:

"UMI-count modeling and differential expression analysis for single-cell RNA sequencing" Wenan Chen, Yan Li, John Easton, David Finkelstein, Gang Wu & Xiang Chen

*Thank you for pointing out this interesting paper. See response to Reviewer 1, comment 1. Also cited in the discussion:*

*While there are certainly opportunities to improve aspects of the negative binomial model (Chen et al 2018, Hafemeister and Satija 2019), it serves as the obvious default model for comparative evaluation of alternative approaches and refinements.*

3 The package and its general use are not thoroughly described. The authors might discuss how one could identify genes that are cell-type specific in their expression based on whether it is zero-inflated (i.e. a gene is ZI when ignoring cell type and is no longer ZI after accounting for it because it's expressed in only one subset of cells.), or other ideas along those lines.

*We agree that some more guidelines for using the scRATE software would be helpful – see response to reviewer 1 comment 4.*

*We want to emphasize that, while the scRATE software was an important tool for us to identify ZI genes and to explain their biological origins, the software itself is not the focus of our manuscript. Our conclusions support an approach to analysis of scRNA-Seq data that does not require scRATE and that can be implemented in standard software packages, e.g., using the NB-GLM as implemented in R/countreg. We don’t wish to downplay the importance of our software as it provides functions that could of interest to some investigators but we don’t see it as an essential step in routine scRNA-Seq analysis. We now emphasized this point in discussion:*

*The scRATE software provides a powerful tool to identify ZI genes in full Bayesian context but we don’t consider it to be an essential step in standard scRNA-Seq workflows. For example, the fastest way to determine that a gene is cell-type specific is to fit the NB-GLM model with and without cell type as a covariate and to compute the likelihood ratio statistic. This can be done using standard GLM software R/countreg (Zeileis et al., 2008). One role for scRATE analysis would be to identify genes that, after accounting for cell type, sex, and other known covariates still appear to be zero inflated. In our analysis of the heart data, we identified a handful of genes, including multiple ribosomal subunit genes that are classified as ZI after accounting for cell type. The scRATE analysis draws our attention to these genes and raises open questions about the possible biological explanations for zero inflation of their count distributions.*

4 The package currently has no vignette on how to run the different functions. I was not able to test it out on my own.

*This was an oversight on our part. We have now provided additional documentation and examples of use for scRATE on the software webpage https://github.com/churchill-lab. The scRATE software is computationally intensive and not intended for use on a laptop. The whole business of getting it to run on a cluster system is challenging and we have tried to simplify the process by containerizing the package. Still, it takes some know-how to run it. See response to comment 2 above and response to reviewer 1 comment 4.*

**Second round of review**

**Reviewer 1**

The authors have addressed all of my comments in a satisfactory manner.

**Reviewer 2**

No additional comments. All comments have been appropriately addressed.
